# Optogenetically Activatable MLKL as a Standalone Functional Module for Necroptosis and Therapeutic Applications in Antitumoral Immunity

**DOI:** 10.1002/advs.202412393

**Published:** 2025-02-08

**Authors:** Da‐Hye Jeong, Seokhwi Kim, Han‐Hee Park, Kyoung‐Jin Woo, Jae‐Il Choi, Minji Choi, Jisoo Shin, So Hyun Park, Myung‐Wook Seon, Dakeun Lee, Jong‐Ho Cha, You‐Sun Kim

**Affiliations:** ^1^ Department of Biochemistry Ajou University School of Medicine Suwon 16499 Republic of Korea; ^2^ Department of Biomedical Science Graduate School of Ajou University Suwon 16499 Republic of Korea; ^3^ Department of Pathology Ajou University School of Medicine Suwon 16499 Republic of Korea; ^4^ Program in Biomedical Science and Engineering Graduate school Inha University Incheon 22212 Republic of Korea; ^5^ Department of Biomedical Sciences College of Medicine Inha University Incheon 22212 Republic of Korea; ^6^ Biohybrid Systems Research Center Inha University Incheon 22212 Republic of Korea

**Keywords:** immunotherapy, MLKL, necroptosis, optogenetics, organoid, RIPK3

## Abstract

Necroptosis plays a crucial role in the progression of various diseases and has gained substantial attention for its potential to activate antitumor immunity. However, the complex signaling networks that regulate necroptosis have made it challenging to fully understand its mechanisms and translate this knowledge into therapeutic applications. To address these challenges, an optogenetically activatable necroptosis system is developed that allows for precise spatiotemporal control of key necroptosis regulators, bypassing complex upstream signaling processes. The system, specifically featuring optoMLKL, demonstrates that it can rapidly assemble into functional higher‐order “hotspots” within cellular membrane compartments, independent of RIPK3‐mediated phosphorylation. Moreover, the functional module of optoMLKL significantly enhances innate immune responses by promoting the release of iDAMPs and cDAMPs, which are critical for initiating antitumor immunity. Furthermore, optoMLKL exhibits antitumor effects when activated in patient‐derived pancreatic cancer organoids, highlighting its potential for clinical application. These findings will pave the way for innovative cancer therapies by leveraging optogenetic approaches to precisely control and enhance necroptosis.

## Graphical abstract

Necroptosis, a key driver of disease progression, holds promise for activating antitumor immunity. The optogenetically controlled necroptosis system, optoMLKL, bypasses upstream signaling, forming functional hotspots in membranes without RIPK3 phosphorylation. This triggers potent immune responses via iDAMP and cDAMP release and exhibits strong antitumor effects in patient‐derived pancreatic cancer organoids, offering a novel avenue for cancer therapy.

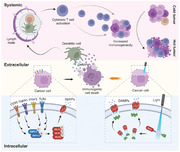

## 1 Introduction

Necroptosis is a form of regulated cell death characterized by distinct molecular mechanisms.^[^ ^1^ ^]^ Central to this process is receptor‐interacting protein kinase 3 (RIPK3), a serine/threonine‐protein kinase that plays a pivotal role in initiating necroptosis.^[^ ^2^ ^]^ RIPK3 phosphorylates and activates its downstream effector, mixed‐lineage kinase domain‐like pseudokinase (MLKL), which then translocates to the plasma membrane, leading to membrane disruption and cell death.^[^ ^3^ ^]^ During this process, damage‐associated molecular patterns (DAMPs) are released, which are key mediators of immune system activation. DAMPs are broadly categorized into constitutive DAMPs (cDAMPs) and inducible DAMPs (iDAMPs) based on their origin and release mechanism.^[^ ^4^ ^]^

cDAMPs such as ATP, high‐mobility group box 1 protein (HMGB1), and calreticulin (CRT) are pre‐existing intracellular molecules that are passively released through channels formed by activated MLKL or from ruptured plasma membranes.^[^ ^4a^ ^]^ These cDAMPs act as “danger signals,” binding to pattern recognition receptors (PRRs) like TLRs and RAGE to promote antigen‐presenting cell (APC) activation and subsequent T‐cell responses.^[^ ^5^ ^]^ ATP activates the P2×7 receptor, leading to inflammasome activation and IL‐1β release,^[^ ^6^ ^]^ while CRT on the cell surface facilitates phagocytosis by dendritic cells.^[^ ^7^ ^]^ Particularly, HMGB1 is significant due to its dual function in immunity. When passively released from necroptotic cells, HMGB1 interacts with TLR4 and RAGE to amplify proinflammatory signaling pathways, enhancing cytokine production and dendritic cell maturation.^[^ ^8^ ^]^

In contrast, iDAMPs are produced or upregulated following specific signaling events triggered by necroptosis. Activated MLKL can translocate to the nucleus, where it stimulates the transcriptional activation of proinflammatory cytokines and chemokines such as IL‐1β, IL‐6, and IL‐8 (CXCL8).^[^ ^9^ ^]^ These iDAMPs enhance the recruitment and activation of immune cells, including macrophages, dendritic cells, and cytotoxic T lymphocytes, creating a robust inflammatory microenvironment.^[^ ^10^ ^]^

The combined release of cDAMPs and iDAMPs during necroptosis not only establishes a proinflammatory milieu but also bridges innate and adaptive immunity, suggesting that necroptosis‐mediated lytic cell death within tumors enhances systemic antitumor immunity.^[^ ^11^ ^]^ Therefore, understanding the mechanisms by which RIPK3 and MLKL contribute to antitumor responses holds significant potential for immunotherapy across various cancers. The controlled induction of necroptosis based on this understanding can be reinterpreted as a therapeutic strategy that not only causes cancer cell damage but also activates antitumor immunity.

Despite advances in our understanding of necroptosis, many aspects of the necroptotic pathway remain unknown. One major challenge is that necroptosis is regulated by complex interactions among multiple signaling pathways, making it difficult to induce necroptosis at a consistent level. Depending on the combination of upstream signals, different biological responses can be triggered, complicating the mechanistic study of necroptosis. For example, inducing the necroptosis pathway in vitro requires complex drug treatments, such as TNF‐α with either SMAC mimetic or cycloheximide, in combination with the pancaspase inhibitor zVAD (herein referred to as TSZ or TCZ). This approach is complicated by ligand‒receptor interactions with other adaptor proteins and the subsequent cascade of reactions in the necroptosis pathway. Additionally, the availability of cell lines expressing RIPK3 is limited because RIPK3 is often silenced through DNA methylation in most cancer cells and tumor tissues.^[^ ^12^ ^]^ To address these limitations, RIPK3 overexpression is often used, but this causes the autophosphorylation and subsequent activation of MLKL, indicating that this approach is insufficient for fully elucidating the complex dynamics of the pathway.^[^ ^13^ ^]^ Thus, methods that can bypass these complex upstream interactions and directly activate the core effectors of necroptosis are clearly needed.

The advent of optogenetic techniques has revolutionized the ability to achieve precise spatiotemporal activation of signaling pathway components, offering advantages over traditional ligand treatments or genetic manipulation.^[^ ^14^ ^]^ This approach allows for the direct activation of key effectors, bypassing complex upstream signaling pathways and enabling consistent signal induction. Optogenetics has proven instrumental in elucidating the complex dynamics of cellular signaling pathways and their dysfunctions in diseases.^[^ ^15^ ^]^ Compared with chemically inducible systems,^[^ ^16^ ^]^ optogenetics offers several advantages, including augmented temporal precision, spatial control, and the capacity to modulate signaling dynamics in live cells with light. This technology can reveal sophisticated aspects of signaling pathways that are not accessible with traditional ligand treatments.^[^ ^17^ ^]^ Although several optogenetically activatable necroptotic platforms have been developed,^[^ ^18^ ^]^ they have primarily been used as cell death‐inducing modules and lack utility as comprehensive analytic tools for pathway dissection. Furthermore, these platforms have largely been restricted to cell lines and xenografts, without validating their efficacy in patient‐derived primary cells and organoids.

Here, we developed optogenetically activatable RIPK3 and MLKL systems to address these limitations. Using these systems, we discovered that necroptosis can be rapidly induced even in the absence of RIPK3. Optogenetically activatable MLKL (optoMLKL) exhibited potent necroptosis independent of its phosphorylation, forming higher‐order oligomers that led to membrane rupture and transcriptional activation. Finally, we applied optogenetically activatable MLKL to induce necroptosis in patient‐derived pancreatic organoids, highlighting its potential for clinical application. Through these approaches, we demonstrated the potential of optogenetics as both a research tool for dissecting the necroptosis pathway and a therapeutic strategy for enhancing antitumor immune responses.

## 2 Results

### 2.1 An Optogenetic Toolkit for RIPK3 Activation Reveals Fine‐Level Induction of Necroptosis

**Figure 1:**
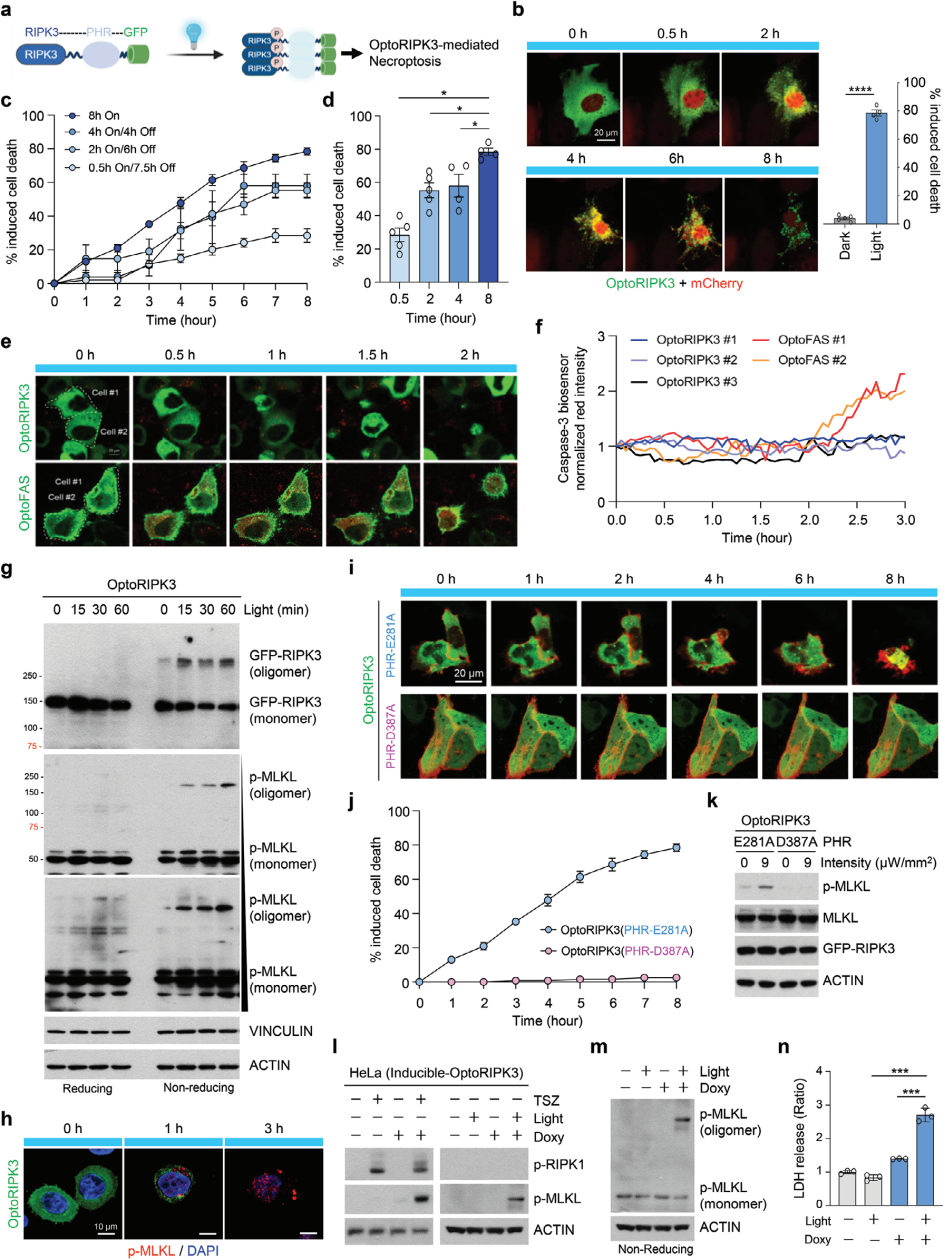
Optogenetically activatable RIPK3 leads to necroptosis without upstream receptor complex. a) Schematic diagram of optogenetically activatable RIPK3 (optoRIPK3), consisting of the full‐length RIPK3 sequence, the PHR domain of cryptochrome 2 (CRY2), and green fluorescent protein (GFP). b) Representative confocal images showing cell death in HeLa cells transfected with optoRIPK3‐GFP following exposure to light. Scale bar, 20 µm. *n* ≥ 80 cells were analyzed in both light and dark group. Mean ± S.E.M.; significance was determined by Mann–Whitney *U* test. c,d) Quantification of cell death levels in HeLa cells transfected with optoRIPK3 under continuous versus transient light stimulation. *n* ≥ 50 cells were analyzed per group. Mean ± S.E.M.; significance was determined by Mann–Whitney *U* test. On, 5 µW mm^−2^ 488 nm illumination; Off, dark incubation. e) Representative confocal images of HeLa cells transfected with optoRIPK3‐GFP and caspase‐3 biosensor undergoing necroptosis, compared to optoFAS‐GFP and caspase‐3 biosensor transfected HeLa cells undergoing apoptosis (green: optoRIPK3‐GFP, optoFAS‐GFP, red: caspase‐3 biosensor). Scale bar, 20 µm. f) Quantification of the red signal of caspase‐3 biosensor activity for the cells shown in e. g) Western blot analysis showing light time‐dependent oligomerization of optoRIPK3, followed by MLKL phosphorylation and oligomerization, in HeLa cells transfected with optoRIPK3‐GFP under reducing and non‐reducing conditions. h) Immunofluorescence analysis of HeLa cells transfected with optoRIPK3‐GFP and exposed to blue light at the indicated time points. (green: optoRIPK3‐GFP; red: p‐MLKL; blue: DAPI). Scale bar, 10 µm. i) Representative confocal images of HeLa cells transfected with optoRIPK3‐GFP containing either PHR(E281A) or PHR(D387A) following exposure to light in a time‐dependent manner. Scale bar, 20 µm. j) Quantification of i. *n* ≥ 50 cells were analyzed in each group. k) Western blot analysis of phosphorylated MLKL in HeLa cells transfected with optoRIPK3 containing either PHR(E281A) or PHR(D387A) upon light stimulation. l) Western blot analysis of optoRIPK3 activation in HeLa cells stably expressing doxycycline (Doxy)‐inducible optoRIPK3‐HA following treatment with TSZ for 4 h or exposure to light for 2 h. m) Western blot analysis revealed that optoRIPK3 induces MLKL phosphorylation and oligomerization under non‐reducing conditions in the cells shown in l. n) LDH release assay was used to measure cytotoxicity in the cells shown in m. *n* = 3 in each group. Mean ± S.D.; significance was determined by unpaired Student's *t*‐test.

To evaluate whether the optogenetic approach enhances our understanding of the necroptosis pathway, we first engineered an optogenetically activatable RIPK3 module comprising the complete RIPK3 sequence, the PHR domain of cryptochrome 2 (CRY2), and green fluorescent protein (GFP) (**Figure** **1**a). The ability of the PHR domain to homo‐oligomerize in response to blue light allows for the close approximation and subsequent phosphorylation of RIPK3, thereby recruiting downstream components of necroptosis.^[^ ^19^ ^]^ Among known PHR mutants that efficiently oligomerize, PHR(E281A) presented the highest degree of cell death induction and the lowest basal rate of cell death^[^ ^20^ ^]^ (Figure S1, Supporting Information). Consequently, we designated the RIPK3‐PHR(E281A)‐EGFP construct optoRIPK3. Activation with 488 nm light successfully induced optoRIPK3 clustering and subsequent death in transfected HeLa cells (Figure 1b).

We then determined the light sensitivity of optoRIPK3 under various light exposure conditions. Although transient light stimulation also induced necroptosis, the efficiency of cell death induction by optoRIPK3 was most pronounced with continuous light exposure. Prolonged light stimulation further increased the level of cell death, which saturated after 8 h of illumination (Figure 1c,d). Induction of cell death by optoRIPK3 was observed even under low‐intensity illumination, revealing RIPK3 oligomerization and downstream MLKL activation, although the efficiency increased with increasing light intensity (Figure S2a, Supporting Information). Necroptotic, rather than apoptotic, cell death induction by optoRIPK3 was confirmed via real‐time imaging with caspase‐3 fluorescent protein exchange biosensors.^[^ ^21^ ^]^ Unlike the optogenetically activatable FAS,^[^ ^15^ ^]^ which induces apoptosis through caspase‐3 activation upon illumination, the cell death induced by the activation of optoRIPK3 did not involve caspase‐3 activity (Figure 1e,f). The TNFα‐mediated necroptosis signaling induction protocol using TSZ,^[^ ^4b^ ^]^ resulted in the phosphorylation/oligomerization of MLKL, which was demonstrated under reducing/non‐reducing conditions or with DTT treatment, which abolished the disulfide bonds and thereby disrupted oligomerization (Figure S2b, Supporting Information). Through this validation assay, we confirmed that activated optoRIPK3 over time induced RIPK3 oligomerization, subsequent phosphorylation, and phosphorylation/oligomerization of MLKL, indicating activation of the necroptotic signaling cascade (Figure 1g). In contrast to TNFα‐mediated necroptosis, optoRIPK3 induced the necroptotic signaling cascade in a rapid and controllable manner on the basis of light intensity (Figure 1g; Figure S2c,d, Supporting Information). Immunofluorescence analysis also revealed that activated optoRIPK3 induced the phosphorylation/oligomerization of MLKL (Figure 1h). The lack of RIPK3 clustering, downstream MLKL activation, or evidence of necroptotic cell death in the non‐oligomerizing PHR mutant D387A further demonstrated that PHR homo‐oligomerization was essential for optoRIPK3 activation, suggesting that optoRIPK3 is specifically controlled in a light‐dependent manner (Figure 1i–k).

An inducible optoRIPK3 cell line, which was transduced with pLenti‐TRE3GV‐RIPK3‐PHR(E281A)‐HA, was successfully generated via dual controllers, doxycycline and blue light, enabling additional fine‐tuned regulation. The phosphorylation/oligomerization of MLKL and robust release of lactate dehydrogenase (LDH) were observed only when inducible‐optoRIPK3‐expressing cells were activated by both doxycycline and blue light (Figure 1l–n; Figure S2e,f, Supporting Information). Collectively, these results demonstrate the successful development of a light‐inducible RIPK3 toolkit capable of inducing necroptotic cell death in a controllable manner.

### 2.2 RHIM‐Independent Oligomerization by optoRIPK3 is Sufficient to Induce Necroptosis

The RIPK3 protein contains a receptor‐interacting protein homotypic interaction motif (RHIM), which allows it to bind with other RHIM‐containing proteins, facilitating oligomerization. This interaction through the RHIM domain is crucial for RIPK3's phosphorylation and activation. Once activated, RIPK3 phosphorylates MLKL, initiating its transition into a functional executioner in necroptosis.^[^ ^22^ ^]^ RIPK3 with a mutated RHIM domain consistently failed to induce adequate oligomerization and activation of downstream components (Figure S3a, Supporting Information). Additionally, the overexpression of full‐length RIPK3 itself induced the oligomerization and phosphorylation of MLKL without any stimulus, whereas truncated RIPK3 lacking the RHIM domain (RIPK3(D328)) did not, indicating that the RHIM domain is essential (Figure S3b, Supporting Information). The requirement of the RHIM domain in response to TNFα‐mediated necroptosis signaling was further demonstrated in inducible optoRIPK3 cells (Figure S3c,d, Supporting Information). Despite the critical role of the RHIM domain in the necroptosis signal, activation with 488 nm light successfully induced RIPK3(D328)‐mediated cell death. However, replacing the functional PHR(E281A) sequence with a nonfunctional PHR(D387A) sequence abolished cell death, suggesting that optoRIPK3 activation independent of RHIM is sufficient to induce cell death (Figure S3e–g, Supporting Information). The efficiency of cell death induction by optoRIPK3(D328), compared with full‐length optoRIPK3, was less pronounced with continuous light exposure. Nonetheless, optoRIPK3(D328) still exhibited light‐specific MLKL phosphorylation/oligomerization (Figure S3h, Supporting Information). The necessity of RHIM‐independent/PHR homo‐oligomerization for optoRIPK3(D328) activation was further demonstrated, as the non‐oligomerizing PHR mutant D387A presented no evidence of RIPK3 clustering or downstream MLKL activation, indicating that optoRIPK3(D328) was specifically controlled in a light‐dependent manner (Figure S3i, Supporting Information). Furthermore, optoRIPK3(D328) induced the release of LDH, which was not observed when RIPK3(D328) remained monomeric under TSZ treatment (Figure S3j,k, Supporting Information). Collectively, these findings suggest that even in the absence of the RHIM domain, the residual portion of RIPK3 induced necroptosis once oligomerization occurred via optogenetic control.

### 2.3 An Optogenetic Toolkit for MLKL Activation Efficiently Induces Necroptosis in a RIPK3‐Independent Manner

**Figure 2:**
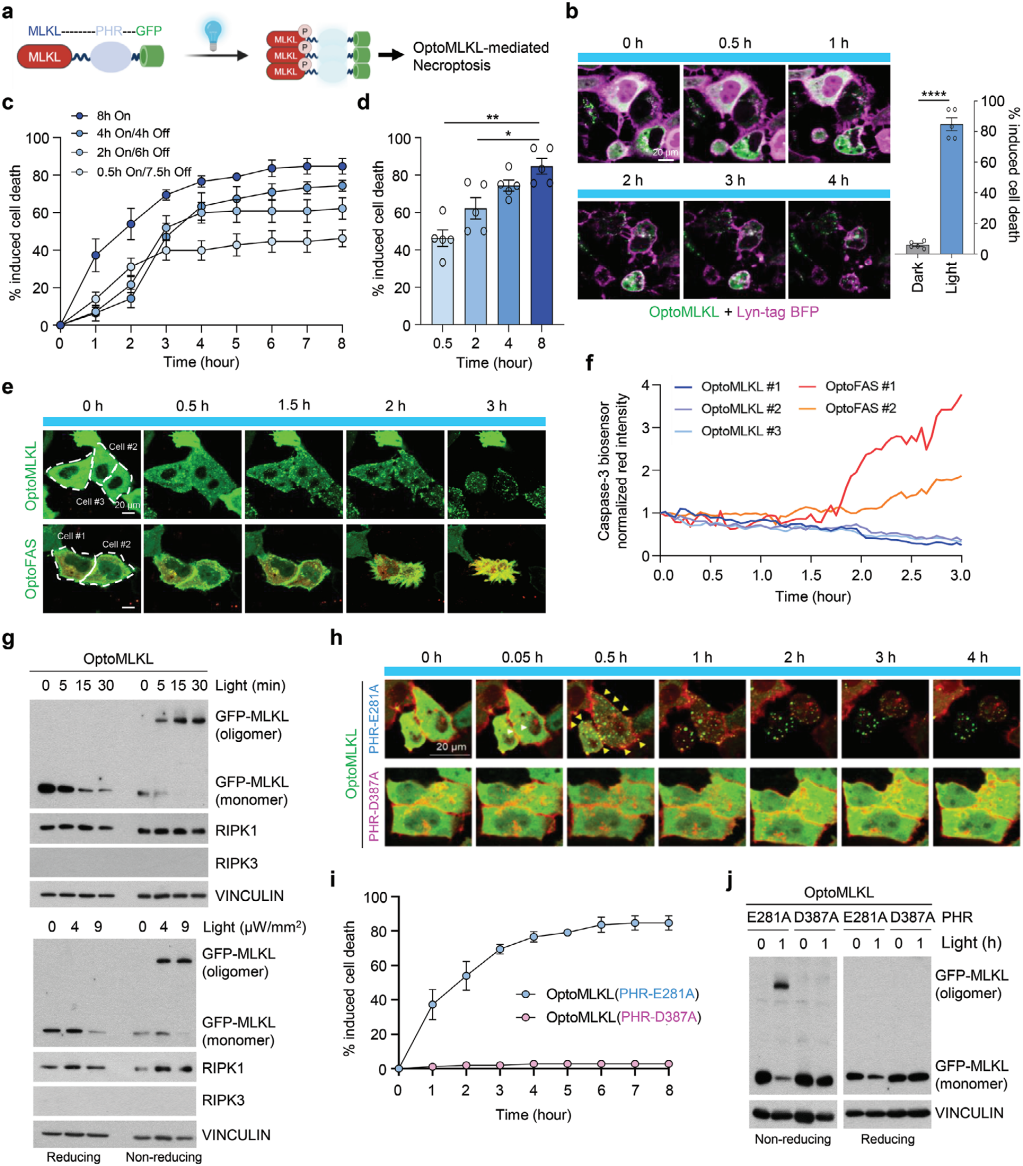
MLKL is rapidly and potently activated by optogenetic modulation. a) Schematic diagram of optogenetically activatable MLKL (optoMLKL), consisting of the full MLKL sequence, the PHR domain of cryptochrome 2 (CRY2), and green fluorescent protein (GFP). b) Representative confocal images of necroptosis induced in optoMLKL‐transfected HeLa cells exposed to light stimulation. *n* ≥ 100 cells were analyzed in both the light and dark groups. Scale bar, 20 µm. Mean ± S.E.M.; significance was determined by Mann–Whitney *U* test. c,d) Quantification of cell death levels in HeLa cells transfected with optoMLKL under continuous versus transient light stimulation. *n* ≥ 50 cells were analyzed per group. Mean ± S.E.M.; significance was determined by Mann–Whitney *U* test. On, 5 µW mm^−2^ 488 nm illumination; Off, dark incubation. e) Representative confocal images of optoMLKL and caspase‐3 biosensor transfected in HeLa cells undergoing necroptosis, compared to optoFAS and caspase‐3 biosensor transfected in HeLa cells undergoing apoptosis. Scale bar, 20 µm. f) Quantification of the red signal of caspase‐3 biosensor activity for the cells shown in e. g) Western blot analysis of HeLa cells transfected with optoMLKL‐GFP showing optoMLKL oligomerization under reducing and non‐reducing conditions. h) Representative confocal images of optoMLKL containing either PHR(E281A) or PHR(D387A) transfected into HeLa cells exposed to light stimulation. Scale bar, 20 µm. i) Quantification of cell death in the cells shown in h. *n* ≥ 50 cells were analyzed in each group. j) Western blot analysis revealing the induction of MLKL oligomerization in a PHR‐dependent manner in HeLa cells transfected with optoMLKL‐GFP containing either PHR(E281A) or PHR(D387A).

We engineered an optogenetically activatable MLKL, optoMLKL, by fusing the PHR domain of CRY2 to the C‐terminus of MLKL and adding GFP, similar to the design of optoRIPK3 (**Figure** **2**a). Surprisingly, blue light stimulation effectively induced cell death in HeLa cells transfected with optoMLKL, even in the absence of RIPK3. This suggests that optoMLKL is capable of inducing necroptosis independently, bypassing the need for RIPK3, which is typically required for MLKL activation in the traditional necroptosis pathway (Figure 2b). Like for optoRIPK3, transient activation also led to cell death, but sustained light stimulation with optoMLKL resulted in the most rapid and significant level of cell death (Figure 2c,d). Upon light stimulation, optoMLKL clustered very rapidly without caspase‐3 activation, suggesting that the mode of cell death was necroptotic (Figure 2e,f). Unlike traditional models that rely on RIPK3‐dependent MLKL oligomerization, the oligomerization of MLKL increased with prolonged illumination and higher light intensity, demonstrating the optogenetic control of this module (Figure 2g; Figure S4a, Supporting Information). Consistent with the lack of activation of caspase‐3 in response to light stimulation, optoMLKL lacked poly (ADP‒ribose)‐polymerase (PARP) cleavage for apoptotic cell death (Figure S4b, Supporting Information). Replacing PHR(E281A) with the nonfunctional PHR(D387A) inhibited MLKL oligomerization, clustering, membrane translocation, and cell death, highlighting the critical role of functional PHR in these processes (Figure 2h–j). Furthermore, dim illumination under ambient light did not activate optoMLKL, suggesting that blue light stimulation effectively activated MLKL to induce cell death (Figure S4c, Supporting Information). The dual‐regulatory module of optoMLKL, combined with a Tet‐on promoter, successfully induced cell death, as shown by crystal violet staining in BT‐549 cell lines lacking endogenous RIPK3 and MLKL (Figure S5a–c, Supporting Information). The TSZ‐induced upstream signal, RIPK1 phosphorylation, was detected in both optoRIPK3‐ and optoMLKL‐expressing cells, indicating that these cells responded properly (Figure S5d, Supporting Information). To further demonstrate that the inducible optoMLKL module can independently trigger cell death without RIPK3 activation, particularly in response to light stimulation, we investigated HeLa cells. Our results confirmed that optoMLKL effectively induces necroptosis in the absence of RIPK3, reinforcing the notion that the optogenetic system bypasses the need for traditional upstream signaling to activate the necroptotic pathway (Figure S5e, Supporting Information).

### 2.4 Hotspot MLKL Oligomerization is Required to Induce Necroptosis in Response to Light Activation

**Figure 3:**
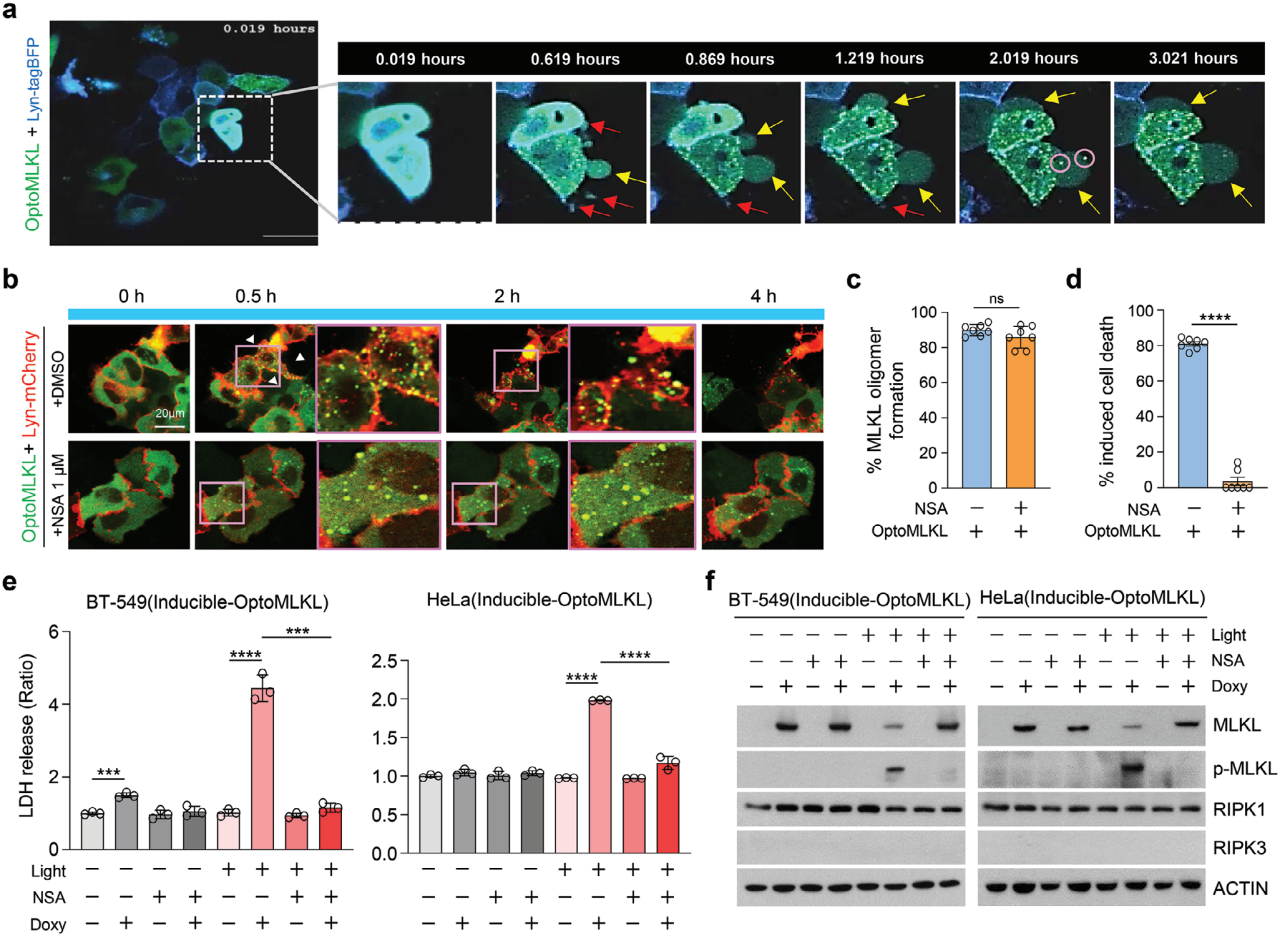
Optogenetically activatable MLKL does not require RIPK3‐dependent phosphorylation to induce necroptosis. a) Real‐time visualization of optoMLKL activation in HeLa cells showing translocation to the plasma membrane following inducible oligomerization. Scale bar, 20 µm. b) Representative confocal images of optoMLKL oligomerization and translocation to the plasma membrane with or without NSA in HeLa cells transfected with optoMLKL‐GFP and Lyn‐mCherry. Scale bar, 20 µm. c) Quantification of the proportion of optoMLKL oligomer formation with or without NSA treatment in the cells shown in b. *n* ≥ 50 cells were analyzed in each group. Mean ± S.E.M.; significance was determined by Mann–Whitney *U* test. d) Quantification of cell death with or without NSA treatment in cells shown in b. *n* ≥ 50 cells were analyzed in each group. Mean ± S.E.M.; significance was determined by Mann–Whitney *U* test. e) LDH release was measured in BT‐549 and HeLa cells stably expressing doxycycline (Doxy)‐inducible optoMLKL, with or without NSA, upon light exposure for 30 min. *n* = 3 cells were analyzed in each group. Mean ± S.D.; significance was determined by unpaired Student's *t*‐test. f) Western blot analysis showing RIPK3‐independent phosphorylation of optoMLKL in BT‐549 and HeLa cells stably expressing doxycycline (Doxy)‐inducible optoMLKL after 30 min of light exposure, in the absence or presence of NSA.

Activated MLKL causes damage to the plasma membrane, and damaged membranes can be repaired through the extrusion of broken membrane parts. In this process, ESCRT‐III is necessary for shedding damaged portions of the plasma membrane during necroptosis, helping to preserve the survival of cells in which MLKL has been activated.^[^ ^23^ ^]^ Consistent with this concept, optoMLKL‐expressing cells exhibited plasma membrane shedding (**Figure** **3**a, red arrow). However, membrane blebbing continued until the integrity of the plasma membrane was completely lost, leading to cell death and disappearance in real‐time visualization (Figure 3a, yellow arrow; oligomerized optoMLKL is shown in pink circles, Figure S6a, continued membrane blebbing is shown by pink arrow in yellow circle, Supporting Information). Necrosulfonamide (NSA), an inhibitor of MLKL, targets cysteine residues to block disulfide cross‐linking, thereby preventing the accumulation of MLKL as micrometer‐sized “hotspots” in the plasma membrane. During TNFα‐mediated necroptosis, after the initial formation of the pronecroptotic oligomer (tetramer), MLKL is thought to further assemble into higher‐order structures which are still unknown the exact nature of these higher‐order oligomers.^[^ ^24^ ^]^ Under these conditions, treatment with NSA did not hinder the formation of the initial pronecroptotic oligomer; however, it remained in the cytosolic portion (Figure S6b, Supporting Information). Although light activation induced MLKL oligomerization, NSA treatment blocked this membrane localization and consequently inhibited cell death (Figure 3b–d). The release of LDH was precisely controlled in a temporally specific manner during optoMLKL activation, but NSA completely inhibited LDH release in both optogenetically activatable BT‐549 and HeLa cells (Figure 3e).

While MLKL oligomerization is necessary but not sufficient for necroptosis. The canonical function of the necrosome is to facilitate the RIPK3‐mediated phosphorylation of MLKL. MLKL consists of an N‐terminal four‐helix bundle (4HB) domain, an intermediary two‐helix “brace” region, and a regulatory pseudokinase domain. The phosphorylation of the pseudokinase domain of MLKL by RIPK3 is a critical step in necroptosis, triggering a conformational change in MLKL. This change releases the autoinhibition of the 4HB domain, allowing MLKL to oligomerize.^[^ ^25^ ^]^ However, RIPK3‐mediated phosphorylation and MLKL oligomerization might be separate processes. This idea is supported by observations showing that optoMLKL was phosphorylated upon light stimulation but that this phosphorylation was blocked by NSA, suggesting that MLKL can form higher‐order oligomers and be phosphorylated independently of RIPK3 (Figure 3f; Figure S6c, Supporting Information). Immunofluorescence using anti‐p‐MLKL antibodies and treatment with the RIPK3 inhibitor GSK’872 confirmed the occurrence of MLKL phosphorylation upon light activation (Figure S6d,e, Supporting Information). Since these phosphorylation sites are known targets of RIPK3, we confirmed the authenticity of this phosphorylation by showing that it disappears after phosphatase treatment (Figure S6f, Supporting Information). To confirm the RIPK3‐independent phosphorylation of optoMLKL, we introduced optogenetically activatable human MLKL into murine cells. Mouse RIPK3 binds only to mouse MLKL but not to its human homolog, suggesting that if the phosphorylation of optogenetically activatable human MLKL is a RIPK3‐dependent event, human MLKL would not be phosphorylated in murine cells. When optogenetically activatable human MLKL was activated by light in mouse 4T1 cells, phosphorylation was detected, and NSA blocked this phosphorylation event, indicating that optoMLKL phosphorylation can occur independently of RIPK3 (Figure S6g, left panel, Supporting Information). Optogenetically activatable mouse MLKL was also phosphorylated upon light activation, but NSA could not block this phosphorylation event (Figure S6g, right panel, Supporting Information). The species specificity of NSA arises from the fact that the cysteine at residue 86 in human MLKL, which is covalently modified by NSA, is replaced by a tryptophan residue in mouse MLKL. We also showed that GSK’872 did not block the phosphorylation of optogenetically activatable mouse MLKL in response to light activation, indicating that the phosphorylation of optoMLKL is a RIPK3‐independent event (Figure S6h, Supporting Information). Based on these data, we speculate that MLKL may mediate signal transduction beyond RIPK3. There are two possibilities for MLKL phosphorylation: either initial pronecroptotic optoMLKL oligomers form hotspots that facilitate the transphosphorylation or autophosphorylation of MLKL, or MLKL may be phosphorylated by other kinases at these hotspots.

### 2.5 Standalone Oligomerization of MLKL is Sufficient for Membrane Translocation and Necroptotic Cell Death

**Figure 4:**
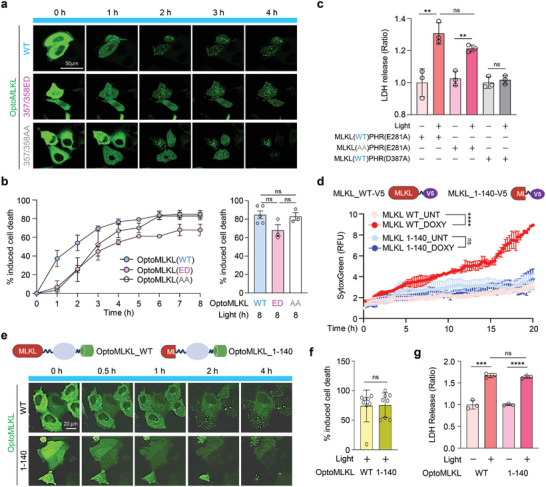
Optogenetically activatable MLKL does not require RIPK3‐dependent phosphorylation to induce necroptosis. a) Representative confocal images of HeLa cells transfected with optoMLKL‐GFP or optoMLKL‐GFP containing phosphorylation site mutants (357/358ED, 357/358AA) at the indicated light times. b) Quantification of cell death in the cells shown in a. *n* ≥ 50 cells were analyzed in each group. Mean ± S.E.M.; significance was determined by Mann–Whitney *U* test. c) LDH release was measured in response to 2 h of light stimulation in HeLa cells transfected with MLKL(WT)‐PHR(E281A), MLKL(T357A/T358A, AA)‐PHR(E281A), or MLKL(WT)‐PHR(D387A). *n* = 3 cells were analyzed per group. Mean ± S.D.; significance was determined by unpaired Student's *t*‐test. d) Cell death in BT‐549 cells stably expressing doxycycline (Doxy)‐inducible MLKL(WT) or MLKL(1‐140) was measured by analyzing the fluorescence intensity of SytoxGreen using Lionheart FX automated microscopy. *n* = 3 in each group. Mean ± S.E.M.; significance was determined by two‐way ANOVA. e) Schematic representation of optoMLKL and optoMLKL(1‐140) (upper panel). Representative confocal images of HeLa cells transfected with optoMLKL‐GFP or optoMLKL(1‐140)‐GFP are shown at indicated light time points (bottom panel). f) Quantitative cell death rates in the cells shown in e. *n* = 8 in each group. Mean ± S.D.; significance was determined by Mann–Whitney *U* test. g) Cytotoxicity was assessed by LDH release in HeLa cells transfected with optoMLKL‐GFP or optoMLKL(1‐140) in response to light for 2 h. *n* = 3 in each group. Mean ± S.D.; significance was determined by unpaired Student's *t*‐test.

Real‐time visualization of optoMLKL activation clearly showed translocation to the plasma membrane and the phosphorylation of optoMLKL. To verify the functional significance of these phosphorylation events on optoMLKL, we introduced double mutations at T357 and S358, which are target residues for phosphorylation, creating either a T357A/S358A (AA form) or T357E/S358D (ED form) mutation. Upon light stimulation, both mutant forms of optoMLKL rapidly clustered and translocated to the plasma membrane, inducing cell death similarly to the wild‐type form. This indicates that the phosphorylation of MLKL is not required for the formation of hotspot oligomers that trigger cell death (**Figure** **4**a–c; Figure S6i–k, Supporting Information). To support this finding, we generated a truncated form of optoMLKL(1‐140) lacking the two‐helix brace region and a regulatory pseudokinase domain. Consistent with previous reports, truncated MLKL(1‐140) without optogenetically activatable region under the Tet‐On system did not induce cell death for 24 h, as monitored by IncuCyte imaging of SytoxGreen‐stained cells (Figure 4d; Figure S6l,m, Supporting Information).^[^ ^23^, ^26^ ^]^ However, real‐time visualization of truncated optoMLKL(1‐140) activation clearly demonstrated that membrane translocation occurred upon inducible oligomerization, with a similar pattern to that observed with the optoMLKL(WT) construct (Figure 4e,f; Figure S6n, Supporting Information). Cell cytotoxicity was observed at similar levels in both optoMLKL(WT) and the truncated form, optoMLKL(1‐140), suggesting that standalone MLKL oligomerization, independent of phosphorylation or other signaling events, was sufficient for membrane translocation and necroptotic cell death (Figure 4g; Figure S6o, Supporting Information). Our data also suggest that the 4HB domain (1‐140) was sufficient to endow MLKL with the ability to disrupt membrane integrity through optogenetic modulation.

### 2.6 Optogenetic Modulation of MLKL Increased Immunogenicity Through the Release of Both iDAMPs and cDAMPs

**Figure 5:**
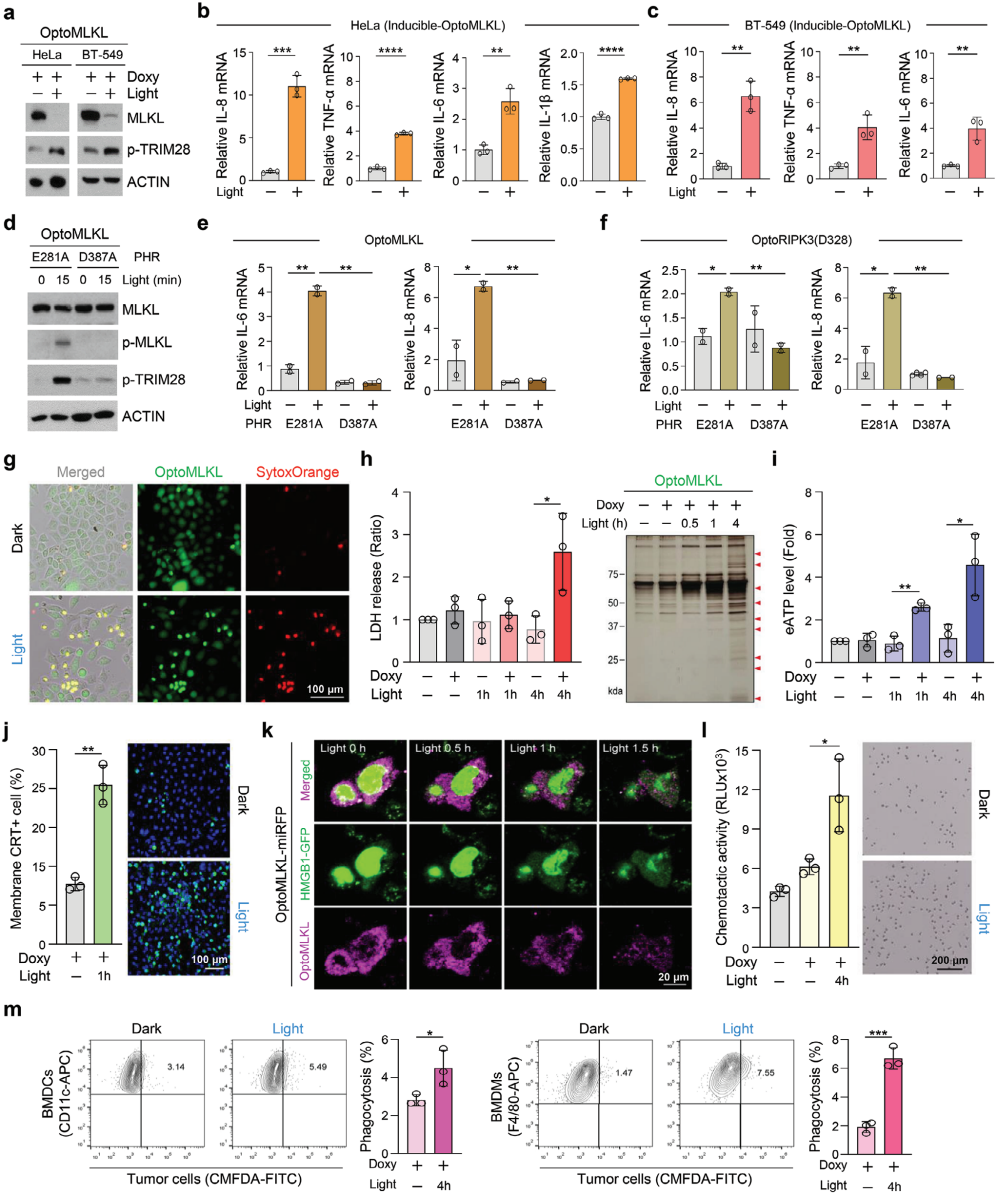
Inducible and constitutive DAMPs were released through the optogenetic activation of MLKL. a) Western blot showing optoMLKL‐dependent phosphorylation of TRIM28 in HeLa and BT‐549 cells following 12 h of optoMLKL induction with doxycycline, followed by exposure to blue light for 3 h. b,c) Inducible DAMPs in HeLa (b) and BT‐549 cells (c) stably expressing doxycycline(Doxy)‐inducible optoMLKL upon illumination were analyzed by qPCR. *n* = 3 in each group. Mean ± S.D.; significance was determined by unpaired Student's *t*‐test. d) Western blot analysis showing PHR‐dependent activation of optoMLKL and TRIM28 in HeLa cells transfected with optoMLKL containing either PHR(E281A) or PHR(D387A). e) Inducible DAMPs were analyzed by qPCR in the cells shown in d. *n* = 3 in each group. Mean ± S.D.; significance was determined by unpaired Student's *t*‐test. f) Inducible DAMPs were analyzed by qPCR in HeLa cells transfected with OptoRIPK3(D328) containing either PHR(E281A) or PHR(D387A). *n* = 3 in each group. Mean ± S.D.; significance was determined by unpaired Student's *t*‐test. g) HeLa cells stably expressing doxycycline (Doxy)‐inducible optoMLKL‐EGFP were sorted using FACS. Cells were exposed to blue light, and membrane rupture was assessed by SytoxOrange staining. Scale bar, 100 µm. h) Doxycycline (Doxy)‐inducible OptoMLKL‐expressing HeLa cells were exposed to blue light, and the levels of LDH released from dying cells were measured (left panel). *n* = 3 in each group. Mean ± S.D.; significance was determined by unpaired Student's *t*‐test. Silver staining was performed to visualize the total protein release under the same treatment conditions (right panel). i) Extracellular ATP levels were measured in the CM of doxycycline (Doxy)‐inducible optoMLKL‐expressing HeLa cells following light stimulation. *n* = 3 in each group. Mean ± S.D.; significance was determined by unpaired Student's *t*‐test. j) After 1 h of blue light exposure, membrane translocation of CRT was examined using immunofluorescence staining in BT‐549 cells expressing optoMLKL‐HA (CRT, green; nuclei, blue). Scale bar: 100 µm. k) Real‐time visualization of HMGB1 release from the nucleus to cytosol and extracellular space by optoMLKL‐miRFP‐transfected HeLa cells upon illumination. Scale bar, 20 µm. l) Quantification of migrated THP‐1 cells in the bottom chamber was conducted by measuring ATP levels (left panel). Representative microscopic images of THP‐1 cells that migrated across the transwell are shown (right panel). Scale bar, 200 µm. m) The phagocytic rate of both BMDCs and BMDMs was assessed using CellTracker CMFDA Green in BT‐549 cells expressing optoMLKL‐HA under light stimulation. *n* = 3 in each group. Mean ± S.D.; significance was determined by unpaired Student's *t*‐test.

We next investigated whether the controlled induction of lytic cell death through optoMLKL activation could lead to the release of DAMPs, a hallmark of immunogenic cell death. Necroptotic dying cells secreted iDAMPs, including various cytokines and chemokines, through an active transcriptional program initiated by the necrosome‐mediated phosphorylation of TRIM28 (Figure S7a, Supporting Information).^[^ ^4c^ ^]^ The selective blockade of RIPK1, RIPK3, or MLKL by Nec‐1, GSK’827, and NSA, respectively, allowed TRIM28 to remain a transcription repressor (Figure S7b, Supporting Information). To induce necroptosis, we utilized three optogenetic toolkits, optoRIPK3, optoRIPK3(D328), and optoMLKL. These systems triggered the phosphorylation of TRIM28, p38, and p65 in response to light stimulation, suggesting that optogenetic modulation may influence transcriptional activity (Figure S7c, Supporting Information). Upon illumination, activation by inducible optoRIPK3 led to the increased transcription of iDAMPs such as IL‐8, TNF‐α, IL‐6, and IL‐1β in both HeLa and MDA‐MB231 cells, both of which lack endogenous RIPK3 (Figure S7d–g, Supporting Information). Notably, the inducible expression of iDAMPs was also observed in HeLa cells with endogenous MLKL and in BT‐549 cells lacking endogenous MLKL upon light activation (**Figure** **5**a–c; Figure S7h,i, Supporting Information). Replacing the PHR domain with the nonfunctional mutant PHR(D387A) did not elicit TRIM28 phosphorylation or the release of iDAMPs for either optoMLKL or optoRIPK3(D328), implying an optogenetic mode of control (Figure 5d–f).

In addition to the induction of iDAMPs, we examined whether optogenetically induced necroptosis leads to the release of typical cDAMPs. Lytic cell death following light exposure was confirmed through silver staining of various dual‐regulatory modules of optoMLKL‐expressing cells (Figure S7j, Supporting Information). After inducing optoMLKL and exposing HeLa cells to light, membrane rupture was indicated by staining with SytoxOrange dye (Figure 5g). The levels of LDH released upon illumination were comparable in conditioned media (CM) collected from dying cells. This demonstrated a time‐dependent increase in intracellular protein release due to light exposure, with peak DAMP release occurring at 4 h (Figure 5h). These results indicate that the cell death induced by optoMLKL activation led to the release of various intracellular substances, resulting in immunogenicity. Indeed, the level of extracellular ATP (eATP), a typical cDAMP during immunogenic cell death,^[^ ^27^ ^]^ significantly increased at both 1 and 4 h after light exposure (Figure 5i). Calreticulin (CRT), a chaperone protein in the endoplasmic reticulum (ER), translocates from the ER to the plasma membrane, where it sends an “eat me” signal to antigen‐presenting cells upon the induction of immunogenic cell death.^[^ ^28^ ^]^ The induction of optoMLKL prompted CRT translocation to the cell membrane 1 h after light exposure (Figure 5j). High mobility group box 1 (HMGB1), a DNA chaperone in the nucleus that maintains chromosomal structure and function, translocates from the nucleus to the extracellular space under stress conditions, thereby regulating inflammation and immune responses in response to infection and tissue damage.^[^ ^29^ ^]^ HMGB1 release from the nucleus was observed starting 1 h after light exposure (Figure 5k); this release was confirmed to be time‐dependent through immunoblotting (Figure S7k, Supporting Information). Finally, we validated the functional activation of innate immunity through controlled lytic cell death via optoMLKL activation. To determine whether CM collected after light exposure, which contains iDAMPs and cDAMPs, could induce chemotaxis in immune cells, we conducted a transwell migration assay with THP‐1 cells. CM was added to the bottom well of a transwell chamber, and substances released from optogenetically induced necroptosis significantly increased the migration of THP‐1 cells (Figure 5l). To assess the antigen‐presenting function of antigen‐presenting cells (APCs) following optogenetically induced necroptosis, we cocultured dying tumor cells exposed to light with bone marrow‐derived dendritic cells (BMDCs) and macrophages (BMDMs). Flow cytometry analysis of phagocytic activity revealed that, compared with that of BMDCs cocultured with control cells, the phagocytic activity of BMDCs cocultured with dying cancer cells induced by optoMLKL activation was significantly greater (Figure 5m). These findings suggest that DAMPs released from dying cancer cells via optogenetically induced necroptosis may create an environment that activates innate immunity within the tumor, potentially enhancing the phagocytic activity of APCs. Overall, necroptosis induced by the activation of optoMLKL increased immunogenicity through the release of both iDAMPs and cDAMPs.

### 2.7 Applications of optoMLKL in Patient‐Derived Pancreatic Cancer Organoids

**Figure 6:**
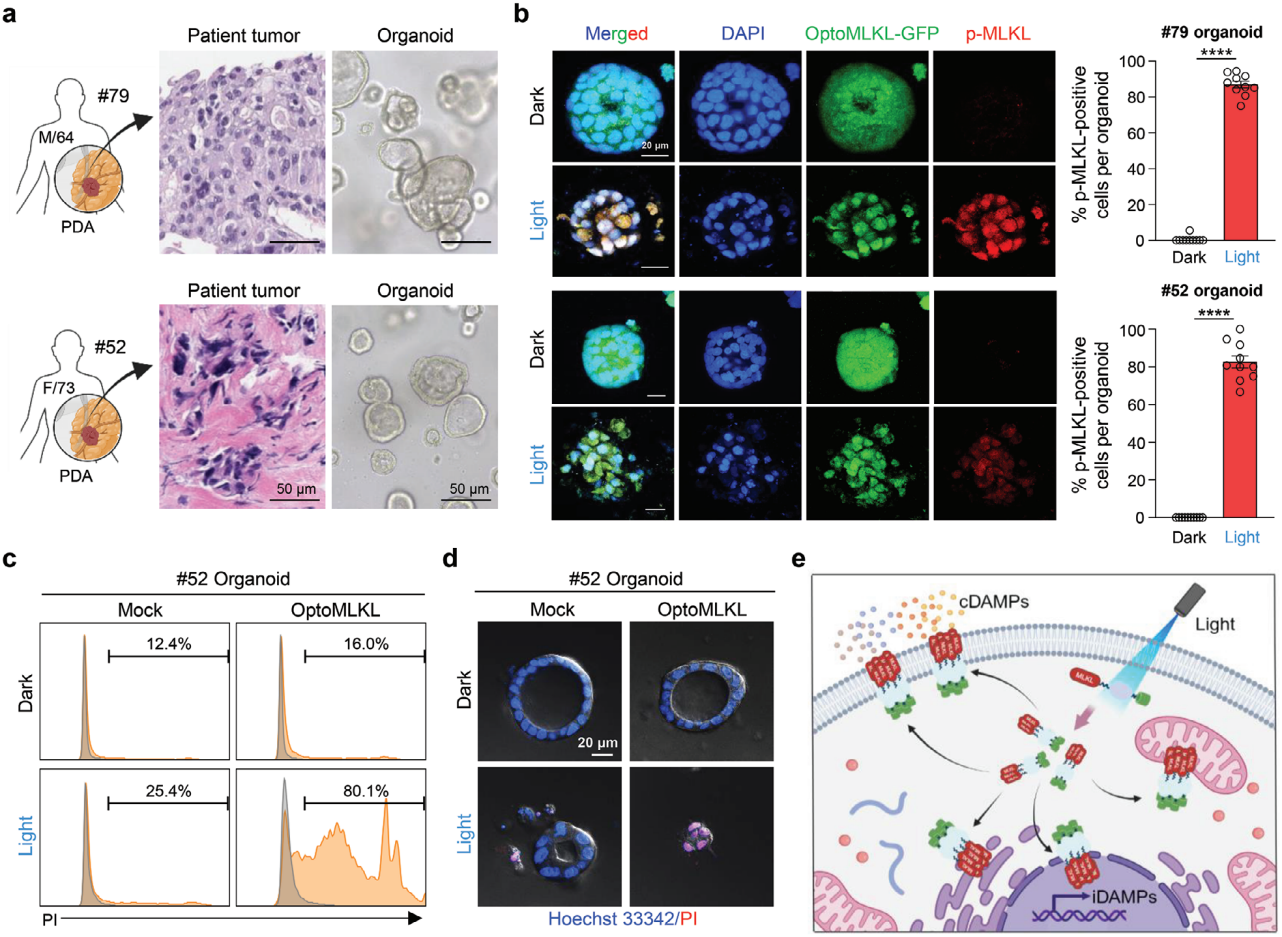
Induction of necroptosis in patient‐derived pancreatic cancer organoids by optoMLKL activation. a) Representative histologic images of patients’ pancreatic cancer (hematoxylin and eosin staining; middle panel) and the corresponding bright‐field images of pancreatic cancer organoids (right panel). Scale bar, 50 µm. b) Representative confocal images of patient‐derived cancer organoids showing induction of MLKL phosphorylation by illumination in optoMLKL‐transduced pancreatic cancer organoid. (#79, upper left panel; #52, lower left panel) and quantification of p‐MLKL‐positive cells per organoid (right panel). Scale bar, 20 µm. Mean ± S.E.M.; significance was determined by Welch's *t*‐test. c) Flow cytometric analysis following propidium iodide (PI) staining of optoMLKL‐transduced #52 organoid upon illumination or dark incubation. d) Representative confocal images of PI‐stained #52 organoids subjected to optoMLKL activation. *n* = 10 in each group. e) Diagram of optoMLKL‐induced necroptosis.

Inducing necroptosis in cancer cells can amplify antitumor immunity and potentially enhance the efficacy of cancer immunotherapy, offering a particularly valuable strategy for treating cancers such as pancreatic cancer, which have an immunosuppressive microenvironment. Pancreatic cancer is known to be highly resistant to conventional therapies, underscoring the urgent need for new treatment strategies.^[^ ^30^ ^]^ To assess the efficacy of optoMLKL in triggering necroptosis in patient‐derived cells, we transduced lentivirus containing pLenti‐EF1α‐optoMLKL into previously established patient‐derived pancreatic cancer organoids (**Figure** **6**a).^[^ ^31^ ^]^ The clinicopathologic characteristics of the corresponding patients are provided in Table S1 (Supporting Information). The organoids were subjected to light stimulation for 3 days, with sessions lasting 6 h per day (Figure S8a, Supporting Information). Upon light stimulation, the necroptotic cell death was visualized and quantified by phosphorylation of optoMLKL. After light activation, significantly high levels of phosphorylated MLKL (p‐MLKL) were detected in all three organoid lines (Figure 6b; Figure S8b,c Supporting Information). Propidium iodide (PI) staining and flow cytometric analysis revealed that a significant portion of the organoids underwent cell death as a result of optoMLKL activation by illumination (Figure 6c; Figure S8d, Supporting Information). OptoMLKL‐transduced organoids exposed to light exhibited a significant increase in the number of PI‐positive cells. In contrast, organoids transduced with optoMLKL but not exposed to light did not exhibit any PI‐positive cells. Likewise, organoids transduced with GFP and exposed to light displayed only a minimal number of PI‐positive cells, thereby confirming the light‐dependent activation of MLKL and induction of cell death in the organoid system. Compared to non‐illuminated, optoMLKL‐transduced organoids or illuminated, GFP‐transduced organoids (mock), optoMLKL‐transduced organoids exposed to light displayed a substantial increase in PI‐positive cells and marked shrinkage, indicative of cell death (Figure 6d). These results suggest the potential application of optoMLKL in human cancers to enhance tumor immunogenicity and augment the efficacy of cancer immunotherapy.

## 3 Discussion

Recent interest in necroptosis has largely stemmed from its potential to increase tumor immunogenicity and stimulate antitumor immunity, making it a promising strategy for cancer immunotherapy.^[^ ^32^ ^]^ While traditional methods that utilize chemicals like TSZ effectively induce necroptosis through the sequential activation of RIPK1, RIPK3, and MLKL, these approaches lack spatial confinement and precise temporal control. Therefore, developing a novel system for necroptosis is crucial for advancing therapeutic applications. Optogenetics has gained significant attention as a tool for achieving precise spatiotemporal control in inducible activation systems.^[^ ^33^ ^]^ Numerous optogenetic systems have successfully induced target protein functions at both the cellular and subcellular levels, facilitating the investigation of their effects and potential applications.^[^ ^34^ ^]^ In our study, we not only validated the capability of our optogenetic system to induce necroptosis but also investigated critical aspects of optogenetic necroptosis systems pertinent to human applications. First, we aimed to dissect signaling pathways by engineering an optogenetic module of signaling pathway components, allowing us to clearly delineate the direct role of individual components. OptoRIPK3 is capable of inducing necroptosis bypassing the need for RHIM domain, which is typically required for the traditional necroptosis pathway in response to a chemical stimulus. The novel finding that optoRIPK3 can induce necroptosis bypassing the RHIM domain suggests that even when full‐length RIPK3 is introduced into cells, it avoids being cleaved by caspases, which often leads to failed cell death induction. This shows that truncated optoRIPK3 can effectively drive necroptosis, increasing the potential for practical applications. Additionally, our data obtained for optoMLKL revealed two important findings: first, the phosphorylation of MLKL by RIPK3 was not necessary to trigger the conformational change required for MLKL activation; second, the oligomerization of MLKL upon illumination was sufficient for MLKL to accumulate as higher‐order “hotspots” in the plasma membrane, leading to membrane rupture (Figure 6e).

The second goal was to confirm that optogenetic necroptosis systems were sufficient to elicit antitumoral immunity. The induction of necroptosis for “cold‐to‐hot tumor transition” has been limited due to the epigenetic suppression of RIPK3 expression in cancer cell lines and primary cancers.^[^ ^13a,b^ ^]^ Attempts to deliver or restore RIPK3 through various approaches have been largely unsatisfactory owing to the difficulty in controlling RIPK3 activity with precise spatial and temporal resolution. Regardless of the RIPK3 status in tumor cells, using optogenetically activatable MLKL can still trigger the release of both cDAMPs and iDAMPs, demonstrating its ability to elicit an immune response independent of upstream RIPK3 signaling. This controlled activation of MLKL offers a targeted approach for modulating antitumoral immunity, presenting significant promise for advancing cancer therapy strategies. The precise and well‐timed release of DAMPs can enhance the immunogenicity of tumors, potentially transforming the tumor microenvironment from an immunosuppressive to an immunostimulatory state, thereby boosting the immune system's ability to fight the tumor.

We demonstrated the clinical relevance of optogenetically induced necroptosis systems by inducing cancer cell death through the activation of optoMLKL in patient‐derived pancreatic cancer organoids. These organoids closely mimic the heterogeneity of actual tumors, providing a more accurate model for testing therapeutic interventions. This fidelity enhances our understanding of treatment efficacy in real‐world scenarios, improving the predictive value of preclinical studies. Moreover, the integration of optogenetics allows precise modulation of necroptotic signaling within a 3D culture system that reflects the complexity of human tumors.^[^ ^35^ ^]^ This innovative approach is essential for determining optimal parameters for necroptosis induction, such as light intensity and duration. By systematically investigating these variables, we can refine clinical protocols and potentially enhance the therapeutic efficacy of necroptosis‐targeting strategies in cancer treatment. Furthermore, the ability to induce necroptosis in a controlled manner opens up possibilities for combination therapies, as shown by the optoSTING system.^[^ ^36^ ^]^ OptoMLKL‐induced necroptosis can also be combined with immune checkpoint inhibitors to enhance the antitumor immune response.

Despite the inherent limitation of light penetration depth into the tissue, which poses a significant challenge to the in vivo application of optogenetics, our optogenetic modules characterized by their exceptional sensitivity are anticipated to function effectively in vivo. Hyeon et al. demonstrated that the cryptochrome 2 PHR domain with an E281A mutation, which is the same light‐responsive module utilized in our study, successfully elicited activation of target cells in the deep brain regions of mice (substantia nigra, dorsal ventral depth of 3.5 mm).^[^ ^37^ ^]^ Notably, in this study, light stimulation was delivered transcranially, suggesting a penetration depth of ≈4 mm when accounting for the thickness of the mouse skull. Similarly, research by Kim et al. corroborated these findings, showing that the cryptochrome PHR domain, including the E281A mutant, effectively induced activation in the thalamic region (dorsal ventral depth of 3.4 mm) using the transcranial approach.^[^ ^38^ ^]^ Furthermore, modifying optogenetic modules to respond to red/far‐red light could facilitate deeper light penetration.^[^ ^39^ ^]^ Implanting light sources in vivo offers another strategy, though further studies are required to adapt and optimize this platform for potential human applications.^[^ ^40^ ^]^

In conclusion, our study demonstrated that the ability to precisely control necroptosis through optogenetics provides a powerful tool for dissecting the molecular mechanisms of necroptosis and for developing new therapeutic strategies. By leveraging the immunogenic properties of necroptotic cell death, optoMLKL could play a pivotal role in enhancing antitumor immunity and improving the efficacy of cancer treatments.

## 4 Experimental Section

### Antibodies and Reagents

Antibodies used for immunoblot and immunofluorescence analysis were as follows: anti‐GFP (Santa Cruz #9996, 1:1000), anti‐HA (Cell Signaling Technology #3724, 1:1000), anti‐Flag (Sigma–Aldrich #F3165, 1:1000), anti‐V5 (Cell signaling Technology #13202, 1:1000), anti‐ACTIN (Santa Cruz #47778, 1:5000), anti‐VINCULIN (Sigma–Aldrich #V9131, 1:5000), anti‐PARP (Cell Signaling Technology #9542, 1:1000), anti‐caspase 3 (Cell Signaling Technology #9662, 1:1000), anti‐RIPK1 (BD #610458, 1:1000), anti‐p‐RIPK1 (Cell Signaling Technology #65746, 1:1000), anti‐RIPK3 (Cell Signaling Technology #13526, 1:1000), anti‐p‐RIPK3 (S227) (Abcam #ab209384, 1:1000), anti‐MLKL (Abcam #184718, 1:1000), anti‐MLKL (Genetex #GTX107538, 1:1000), anti‐p‐MLKL (Abcam #ab187091, 1:1000), anti‐p‐TRIM28 (S473) (BioLegend #654102, 1:2000), anti‐HMGB1 (Abcam #ab18256, 1:1000), anti‐HMGB1 (Cell Signaling Technology #6893S, 1:2000), anti‐EGFP (Abcam #184601, 1:1000), Alexa Fluor 488 anti‐Calreticulin (Abcam #ab196158, 1:500). TNF‐α and zVAD were purchased from R&D Systems. The SMAC mimetic (#LCL‐161) was obtained from Adooq Bioscience. Necrostatin‐1 was from Sigma–Aldrich. NSA and GSK’872 were purchased from Merck. Doxycycline was purchased from Clontech (#631310).

### Cell Culture

293 T, HT‐29, HeLa, BT‐549 and MDA‐MB231 cells were grown in Dulbecco's modified Eagle's medium (DMEM; Gibco #11965092) supplemented with 10% fetal bovine serum (FBS; Invitrogen) and penicillin/streptomycin (Gibco #15140122). All cells were cultured in 37  °C, 5% CO_2_ incubators. Cell lines were confirmed to be contamination‐free using an e‐MycoTM Mycoplasma PCR detection kit (iNtRON). Ninety‐six–well plates (Ibidi #89626) were used for cell culture and live‐cell imaging.

### Plasmid and Cloning

To construct RIPK3‐PHR(E281A)‐EGFP, the human RIPK3 full sequence^[^ ^41^ ^]^ was amplified by polymerase chain reaction (PCR) and inserted into Lyn‐FAS‐PHR(E281A)‐EGFP^[^ ^42^ ^]^ by using restriction enzymes NheI/HindIII, replacing the Lyn‐FAS sequence. RIPK3‐PHR(E490G)‐EGFP, RIPK3‐PHR(WT)‐EGFP, and RIPK3‐PHR(D387A)‐EGFP were generated by replacing PHR(E281A) sequence by PHR(E490G), wild type PHR, and PHR(D387A)^[^ ^42^ ^]^ using restriction enzyme cloning with HindIII/AgeI, HindIII/AgeI, and XmaI/AgeI, respectively. pLenti‐TRE3GV‐RIPK3‐PHR(E281A)‐HA was constructed from pLenti‐CMV‐TRE3G‐NeoGFP‐Progerin (Addgene #118710), inserting RIPK3‐PHR(E281A)‐HA by using ClaI/AgeI restriction enzymes. RIPK3(D328)‐PHR(E281A)‐EGFP and RIPK3(D328)‐PHR(D387A)‐EGFP were generated with backbone vectors of RIPK3‐PHR(E281A)‐EGFP and RIPK3‐PHR(D387A)‐EGFP by replacing the full‐length RIPK3 sequence into the RIPK3(D328) sequence with NheI/HindIII and XmaI/AgeI restriction enzyme digestion, respectively. MLKL‐PHR(E281A)‐EGFP was constructed with the PCR‐amplified human MLKL sequence,^[^ ^41^ ^]^ inserted into Lyn‐FAS‐PHR(E281A)‐EGFP by using restriction enzymes NheI/EcoRI, replacing the Lyn‐FAS sequence. MLKL‐PHR(E490G)‐EGFP, MLKL‐PHR(WT)‐EGFP, and MLKL‐PHR(D387A)‐EGFP were engineered by replacing PHR(E281A) sequence by PHR(E490G), wild type PHR, and PHR(D387A) by EcoRI/AgeI restriction enzyme clonings. pLenti‐TRE3GV‐MLKL‐PHR(E281A)‐HA was generated from pLenti‐CMV‐TRE3G‐NeoGFP‐Progerin (Addgene #118710), inserting MLKL‐PHR(E281A)‐HA by using ClaI/AgeI restriction enzymes. Mouse MLKL‐PHR(E281A)‐EGFP was constructed by replacing the human MLKL sequence of MLKL‐PHR(E281A)‐EGFP into the PCR‐amplified mouse MLKL sequence by AgeI/NotI restriction enzyme cloning. pLenti‐EF1a‐MLKL‐PHR(E281A)‐EGFP was constructed by Gibson Assembly Cloning (New England Biolabs), which was used to replace EGFP with PCR‐amplified MLKL‐PHR(E281A)‐EGFP in the pLenti‐EF1a‐SPdCas9‐EGFP‐2A‐Blast vector (Addgene #71215). The list of primers utilized for cloning is summarized in Table S2 (Supporting Information).

### Plasmid Transfection

Transfection of the cell lines was conducted using Lipofectamine LTX (Invitrogen #94756) or polyethylenimine (PEI; Polysciences #Q‐2476‐2) according to the manufacturer's instructions. Briefly, cells were seeded in culture dishes, and transfection reagents were mixed with plasmid DNA in Opto‐MEM (Gibco #31985). The mixture was incubated for 15 min at room temperature, to allow complex formation. After the transfection, cells were incubated under culture conditions for 16 h.

### Lentivirus Production and In Vitro Viral Transduction

For lentivirus production, the transfer plasmid, Δ8.9 plasmid, and vesicular stomatitis virus G (VSVG) plasmid were diluted in Opti‐MEM in a 4:3:1 ratio. Lipofectamine 2000 (Invitrogen) was added to achieve a 1:5 mixture of DNA (µg) to Lipofectamine 2000 (µL) in other tube. After a 5 min incubation at room temperature, the solutions were mixed and incubated for an additional 15 min at room temperature. The mixture was then distributed onto 15 cm dishes containing HEK293T cells at 75%–80% confluency. The culture medium was completely replaced by 4 h post‐transfection. At 48 h after the transfection, the culture supernatant was collected, centrifuged at 1500 rpm for 15 min at 4 °C, and filtered through a 0.45 µm filter. HeLa, BT‐549, and MDA‐MB231 cells were incubated in a lentivirus‐containing medium supplemented with polybrene. 48 h following infection, the cells were transferred to 10 cm dishes, and puromycin was applied for 48 h for selection. In the established stable cell lines, optogenetic constructs were expressed with doxycycline.

### Live‐Cell Imaging and Photoactivation

Live‐cell imaging was conducted using a Nikon AXR confocal microscope equipped with CFI Plan Apo objectives at a magnification of ×60. Multicolor images were captured with lasers at wavelengths of 405, 488, 561, and 647 nm. Temperature (37 °C) and CO_2_ concentration (10%) were maintained using a Tokai Hit automatic thermocontrol system (Tokai Hit) installed on the microscope stage. Photoactivation was performed with a 488 nm laser transmitted through a Galvano scanner, integrated into a hybrid confocal scan head with a high‐speed selector (Nikon). For most in vitro experiments, a light intensity of 5 µW mm^−^^2^ and a frequency of 1 s every 3 min (duty cycle, 0.55%) were employed for activation, unless otherwise noted. The stimulation area was adjusted using data obtained from the Nikon imaging software (NIS‐elements AR 64‐bit version 4.10, Laboratory Imaging).

### Light‐Emitting Diode (LED) Stimulation

Cells were illuminated using a TouchBright W‐96 LED Excitation System (Live Cell Instrument). The system allowed for adjustment of the LED power intensity to provide 470 nm light at varying intensities from 0 to 20 µW mm^−^^2^. In most experiments, the cells were exposed to a light intensity of 9 µW mm^−^^2^ with a duty cycle of 33% (1 s of light followed by 2 s of darkness).

### Cell Cytotoxicity and Viability Assay

Cell cytotoxicity and viability were assessed using LDH release assay, crystal violet staining, and Cell Counting Kit‐8 assay. For LDH release measurement, cell supernatant was analyzed using the CytoTox 96 Non‐Radioactive Cytotoxicity Assay kit (Promega #G1780) according to the manufacturer's instructions. Absorbance was measured at 490 nm. For crystal violet staining, cells were fixed with 4% paraformaldehyde (PFA; Sigma–Aldrich #158127), stained with 0.5% crystal violet (VWR #0528), and washed three times with deionized water. The stain was solubilized in methanol (Merck #106009), and absorbance was measured at 590 nm using a POLARstar OPTIMA Multidetection Microplate Reader. For cell viability assessment, in Figure S2a (Supporting Information), Cell Counting Kit‐8 (Dojindo Molecular Technologies #CK04‐01) was used according to the manufacturer's instructions. Cells were incubated in a medium containing 10% Cell Counting Kit‐8 solution, and absorbance was measured at 450 nm using a VersaMax microplate reader (Molecular Devices).

### Reducing and Non‐Reducing Western Blot Analysis

For reducing western blotting, the cells were lysed in M2 lysis buffer (20 mm Tris at pH 7, 0.5% NP‐40, 250 mm NaCl, 3 mm EDTA, 3 mm EGTA, 2 mm DTT, 0.5 mm PMSF, 20 mm β‐glycerol phosphate, 1 mm sodium vanadate, and 1 mg mL^−1^ leupeptin). For non‐reducing western blotting, the cells were lysed in M2 lysis buffer without DTT. Cell lysates were mixed with SDS sampling buffer in the absence or presence of β‐mercaptoethanol, resolved by SDS‐PAGE and immunoblotting, and visualized by enhanced chemiluminescence (Pierce ECL Western Blotting Substrate, #32106).

### BN‐PAGE Analysis

To extract the cytosol fraction, NativePAGE Sample Buffer containing 0.025% DDM was used. The non‐soluble region was lysed with 1% DDM buffer (Invitrogen #BN2008). Both fractions were analyzed by Bis‐Tris Native PAGE (Invitrogen #BN1001) and immunoblotting according to the manufacturer's instructions. The blots were visualized using enhanced chemiluminescence (Pierce ECL Western Blotting Substrate, #32106).

### Quantitative Real‐Time‐PCR

RNA was extracted^[^ ^43^ ^]^ using the TRIzol reagent (Life Technologies #15596018). Total RNA (1 µg) from each sample was used for cDNA synthesis using MMLV reverse transcriptase (MGmed #MR10601). Equal amounts of cDNA product were used in real‐time PCR with GoTaq qPCR Master Mix (Promega #A6001). Gene expression was normalized to that of Actin. Real‐time PCR was performed on CFX Connect. The list of utilized primers for RT‐PCR was summarized in Table S3 (Supporting Information).

### Immunocytochemistry

For immunocytochemical (ICC) staining,^[^ ^44^ ^]^ cells were washed twice with DPBS, fixed in 4% PFA for 15 min at room temperature, and permeabilized with 0.25% Triton X‐100 for 7 min. After incubation in a blocking buffer for 30 min, the cells were incubated overnight at 4 °C with primary antibodies. Then, they were incubated with the following Alexa Fluor secondary antibodies (Invitrogen) for 2 h at room temperature: 594‐conjugated rabbit (#A11037), 594‐conjugated mouse (#A21125), and 488‐conjugated rabbit (#A11008), 488‐conjugated mouse (#A11001). A mounting medium containing DAPI (Vector Laboratories #94010) was used for counterstaining.

### Silver Staining

For silver staining, cells were replaced with serum‐free media. After exposure to blue light, the supernatant was centrifuged at 450 g for 5 min, and 400 µL of the supernatant was collected. The samples were diluted in sample buffer and boiled for at 95 °C for 5 min, and subjected to SDS‐PAGE. The gel was fixed overnight in fix solution (DW, 30% ethanol, 10% acetic acid) and silver staining was performed using the Silver Stain Kit (ELPIS‐Biotech #EBP‐1051) according to the manufacturer's instructions.

### Calreticulin Immunofluorescence Staining

Cells were seeded in a 6‐well plate and a 15 mm^2^ coverslip. Immunocytochemistry was performed by cells with Recombinant Alexa Fluor 488 anti‐Calreticulin (Abcam #ab196158, 1:500) in 3% BSA overnight 4 °C. Images were captured using a Nikon DS‐Ri2 camera, and CRT was quantified using ImageJ software (v1.53e).

### Measurement of Extracellular ATP Assay

Cells were seeded in a 96‐well culture plate and pre‐treated with the eATP inhibitor (Sigma–Aldrich #ARL67156, 300 µm) for 30 min. After exposure to blue light for a specified period, the supernatant was centrifuged at 1500 RPM for 5 min, and 50 µL of the collected supernatant was transferred to a 96‐well white plate. CellTiter‐Glo reagent (Promega #G9241) was added to each well and incubated at room temperature for 5 min. Luminescence was then measured to assess extracellular ATP levels.

### Measurement of HMGB1 Secretion (CM Concentration)

Cells were seeded in a 12‐well culture plate. After exposure to blue light, CM was centrifuged at 450 g for 5 min to remove debris. The CM was concentrated using an Amicon Ultra‐0.5 Centrifugal Filter Unit (Millipore #UFC500396) at 17500 RPM for 30 min, followed by a reverse spin step for recovery. Each sample was prepared with the same volume of loading buffer (DW, 50 mm Tris‐HCl pH 6.8, 100% glycerol, 20% SDS, 2‐mercaptoethanol, bromophenol blue). Cells were lysed using 150 µL of urea buffer (Distilled water: 5 m urea: 10% SDS = 1:1:1 ratio), scraped, and transferred to a microtube. The transferred cells were sonicated twice, and protein concentration was measured using BCA reagent. Each sample was boiled for 5 min at 95 °C. Denatured protein samples were separated by 12% SDS‐PAGE and transferred to a nitrocellulose membrane. Transferred proteins were blocked using 5% (w/v) skimmed milk/PBST (DW, 20X PBS, 0.1% Tween 20) for 30 min and the membrane was washed three times with PBST for 10 min. The primary antibody for human HMGB1 (Cell Signaling Technology #6893S, 1:2000) was incubated at 4 °C overnight, followed by incubation with anti‐rabbit HRP (Cell Signaling Technology #7074P2, 1:10000) at room temperature for 1 h. The membrane was detected using the Pierce ECL Western Blotting Substrate. Image acquisitions were performed using the Fusion solo S (Vilber Lourmat Deutschland GmbH, Germany).

### THP‐1 Migration Assay

For trans‐well migration assays, 24‐well plates equipped with trans‐well chambers featuring 5 µm pores (Corning #3421) were used. THP‐1 cells were subjected to a cell‐down process, suspended in FBS‐free media, and incubated at 37 °C for 4 h to induce starvation. Starved THP‐1 cells were resuspended in media containing 2% FBS and seeded in the upper chamber at a density of 5 × 10^5^ cells in 200 µL per well. The lower chambers were filled with 600 µL of media containing concentrated conditioned media with the same composition as the upper chamber. Cells were allowed to migrate for 6 h at 37 °C. After migration, THP‐1 cells that migrated to the lower chamber were collected, subjected to a cell down process, and resuspended in RPMI media, and cell viability was assessed using the CellTiter‐Glo reagent kit in accordance with the manufacturer's instructions.

### Generation of Murine Bone Marrow DCs, and DMs

Bone marrow cells were harvested from the femurs and tibias of C57BL/6 mice, flushed, and filtered through a 40 µm filter to remove aggregates and impurities. After centrifugation at 450 g for 5 min, red blood cells were lysed using RBC lysis buffer for 5 min at room temperature. Cells were resuspended in RPMI supplemented with murine GM‐CSF (Peprotech #214‐14‐100UG, 20 ng mL^−1^) and murine IL‐4 (Peprotech #315‐03‐100UG, 20 ng mL^−1^) for BMDCs while BMDM cells were cultured in RPMI containing GM‐CSF alone. BM cells were seeded onto bacterial petri dishes. On day 3, an equal volume of fresh media containing GM‐CSF and IL‐4 was added to the BMDCs, and only media containing GM‐CSF was added to the BMDMs. On day 6, half of the culture medium was replaced with fresh medium containing the same concentration of cytokines. On day 8, the cells were harvested by gentle pipetting for use in experiments.

### Phagocytosis Assay

Cells were stained in suspension with CellTrackerTM Green CMFDA (Invitrogen #C7025, 1 µm) for 30 min at 37 °C, then seeded in a 60 mm culture dish. The next day, cells were either exposed to light at a wavelength of 488 nm for 4 h or left untreated for the same duration. After 4 h, the cancer cells were co‐cultured with BMDCs or BMDMs at a 1:1 ratio for 2 h at 37 °C. BMDCs were stained with APC‐conjugated CD11c (BioLegend #117310, 1:50) and BMDMs were stained with APC‐conjugated F4/80 (BioLegend #157306, 1:100) for 30 min at 4 °C. The phagocytosis of cancer cells was analyzed by flow cytometry.

### Organoid Culture

Pancreatic cancer organoids from endoscopic ultrasound‐guided fine‐needle biopsy sample of two patients (patients #52, #70, and #79) following methods described in a previous study (NCT05571956).^[^ ^38^, ^39^ ^]^ Written informed consent was obtained from all patients, and the study protocol was approved by the institutional review board of Ajou University Hospital (AJIRB‐BMR‐20‐222). This study is registered at https://clinicaltrials.gov as NCT05571956. Briefly, organoids were embedded in growth factor reduced (GFR) Matrigel and maintained using culture medium consisting of DMEM/F12 medium supplemented with GlutaMAX (Thermo Fisher Scientific), penicillin/streptomycin (Thermo Fisher Scientific), B27 (Thermo Fisher Scientific), N‐acetyl‐L‐cysteine (Sigma–Aldrich, 1 mm), Wnt3a‐conditioned medium (50% v/v), RSPO1‐conditioned medium (R&D Systems, 10% v/v), recombinant noggin protein (PeproTech, 100 ng mL^−1^), recombinant epidermal growth factor protein (EGF; PeproTech, 50 ng mL^−1^), gastrin (Sigma–Aldrich, 10 n *m*), recombinant fibroblast growth factor 10 protein (FGF10; PeproTech, 100 ng mL^−1^), nicotinamide (Sigma–Aldrich, 10 mm), and A83‐01 (Tocris, 0.5 µm).

### Light Stimulation and ICC of Organoids

Pancreatic cancer organoids were transduced either by pLenti‐EF1a‐optoMLKL‐GFP virus or control GFP lentivirus using the polybrene (Santa Cruz Biotechnology). Following transduction, organoids were stimulated with LED light at an intensity 20 µW mm^−^^2^ for 3 days, with a cycle of 1 s on and 2 s off, totaling 6 h of stimulation per day. Control organoids were maintained in the dark condition. For intracellular staining for phosphorylated MLKL, organoids were fixed using 1% PFA with 0.1% glutaraldehyde and washed three times with PBS containing 10 mm NaBH_4_. Organoids were blocked and permeabilized with 1% BSA and 0.2% Triton X‐100 at room temperature for 1 h. Staining was performed using anti‐p‐MLKL and anti‐EGFP antibodies, followed by incubated with a secondary antibody. After three washes, images were obtained by using Nikon AXR confocal microscope (Nikon) with the 20x CFI Pan Apochromat Lambda objective. To stain dead cells following optoMLKL activation, organoids transduced with lentivirus were stimulated by optoMLKL by LED exposure for 3 days. After a day, organoids were stained with propidium iodide (Sigma‐Aldrich, 1 µg mL^−1^), and Hoechst (Thermo Fisher Scientific #33342) without fixation or permeabilization process. FACS analysis was performed to evaluate the viable cells by using Canto II (BD Bioscience) and FlowJo (v10). Additionally, the fluorescence images were captured using Nikon AXR confocal microscope.

### Quantification and Statistical Analysis

All confocal images were analyzed using Nikon imaging software (NIS‐elements AR 64‐bit version 4.10, Laboratory Imaging). To calculate the induced cell death rate, the number of cells showing morphological features of necroptosis was counted from more than five randomly selected imaging fields for each experimental condition. In most images, the region of interest (ROI) was selected using the “Automated ROI” function of the Nikon imaging software. The normalized C/N ratio of the caspase biosensor was measured using the “Time Measurement” tool of the imaging software. Statistical analyses were performed using GraphPad Prism 10.3.1 (GraphPad Software). Statistical significance was determined using a two‐tailed unpaired Student's *t*‐test with unequal variance, Mann‐Whitney *U* test, Welch's *t*‐test, or one‐way analysis of variance (ANOVA) followed by Tukey's or Fisher's post hoc tests for multiple comparisons. Data are presented as Mean ± S.E.M. or S.D.; significance indicated as: ns (not significant), ^*^ *p* < 0.05, ^**^ *p* < 0.01, ^***^ *p* < 0.001, and ^****^ *p* < 0.0001.

### Reporting Summary

Further information on research design was available in the Nature Portfolio Reporting Summary linked to this article.

## Conflict of Interest

The authors declare no conflict of interest.

## Author Contributions

J.‐H.C. and Y.‐S.K. conceived the idea and designed experiments. D.‐H.J. performed the most experiments and analyzed the data. K.‐J.W. and S.K. designed plasmid vectors and conducted live cell imaging. J.‐I.C. and S.H.P. performed the organoid experiment and analyzed the data. M.C., J.S. performed experiments on DAMPs release and innate immunity. S.K. wrote an initial draft of the paper. S.K., D.L., J.‐H.C., and Y.‐S.K. edited the manuscript. Y.‐S.K. supervised the study and took responsibility for all of the data. All authors reviewed the final version of the manuscript.

## Supporting information



Supporting Information

## Data Availability

The data that support the findings of this study are available from the corresponding author upon reasonable request.
