# Supplementary material for: Optogenetically Activatable MLKL as a Standalone Functional Module for Necroptosis and Therapeutic Applications in Antitumoral Immunity
Source: Adv Sci (Weinh). 2025 Feb 8;12(13):2412393. doi: 10.1002/advs.202412393 (PMC11967802; doi:10.1002/advs.202412393)
Supplement: Supplementary file 1 — Supporting Information [file ADVS-12-2412393-s001.docx]

**Supplementary Information for**

**Optogenetically activatable MLKL as a Standalone Functional Module for Necroptosis and Therapeutic Applications in Antitumoral Immunity**

Da-Hye Jeong^1,2^, Seokhwi Kim^2,3^, Han-Hee Park^1,2^, Kyoung-Jin Woo^2^, Jae-Il Choi^3^, Minji Choi^6^, Jisoo Shin^6^, So Hyun Park^3^, Myung-Wook Seon^1,2^, Dakeun Lee^2,3^, Jong-Ho Cha^4, 5, 6^, You-Sun Kim^1,2,*^

^1^Department of Biochemistry, Ajou University School of Medicine, Suwon, 16499 Republic of Korea,

^2^Department of Biomedical Science, Graduate School of Ajou University, Suwon, 16499 Republic of Korea

^3^Department of Pathology, Ajou University School of Medicine, Suwon, 16499, Republic of Korea

^4^Department of Biomedical Sciences, College of Medicine, Inha University, Incheon, 22212, Republic of Korea

^5^Biohybrid Systems Research Center, Inha University, Incheon, 22212, Republic of Korea

^6^Program in Biomedical Science and Engineering, Graduate school, Inha University, Incheon, 22212, Republic of Korea

^*^Correspondence: You-Sun Kim, Ph.D.

Department of Biochemistry, Ajou University School of Medicine

Department of Biomedical Science, Ajou University Graduate School of Medicine

164 Worldcup-ro, Yeongtong-gu, Suwon, 16499, Republic of Korea

Tel: +82-31-219-4509, E-mail: [yousunkim@ajou.ac.kr](mailto:yousunkim@ajou.ac.kr)

ORCID: 0000-0001-6488-3015

This file includes:

This file includes:

Supplementary Figure S1 (Efficiency and basal rate of inducing cell death in PHR mutants, page 9)

Supplementary Figure S2 (Necroptosis induction of optoRIPK3 with varying light intensities, page 10)

Supplementary Figure S3 (Optogenetically activatable RIPK3, lacking RHIM domain, is capable of inducing necroptosis, page 11)

Supplementary Figure S4 (Optogenetic modulation of MLKL induces its oligomerization, page 12)

Supplementary Figure S5 (Optogenetic activation of MLKL induces necroptosis regardless of the presence of RIPK3, page 13)

Supplementary Figure S6 (Phosphorylation of MLKL is not required for necroptosis induction via optogenetic modulation, page 14)

Supplementary Figure S7 (DAMPs released upon optogenetic activation of RIPK3 and MLKL, page 15)

Supplementary Figure S8 (OptoMLKL activation in patient-derived pancreatic cancer organoid, page 16)

Supplementary Table 1 (Clinicopathologic characteristics of the patient-derived organoids, page 17)

Supplementary Table 2 (List of primers for plasmid vector construction, page 18)

Supplementary Table 3 (List of primers for real-time PCR, page 19)

**Supplementary data information**

**Supplementary Figure S1. | Efficiency and basal rate of inducing cell death in PHR mutants.**

Quantification of cell death levels and rates in HeLa cells expressing RIPK3 PHR mutants under light stimulation.

**Supplementary Figure S2. | Necroptosis induction of optoRIPK3 with varying light intensities.**

**a,** Cell viability was assessed using Cell Counting Kit-8 in optoRIPK3-transfected HeLa cells exposed to varying light intensities. Absorbance at 450 nm measured to estimate cell death. Mean ± S.E.M.; significance was determined by Mann-Whitney test. **b,** Western blot analysis showing the phosphorylation and oligomerization of MLKL in TSZ-treated HT-29 cells under reducing, non-reducing, or DTT treatment conditions. **c,** Western blot analysis showing optoRIPK3 oligomerization under light intensity-dependent exposure, leading to MLKL oligomerization in HeLa cells transfected with optoRIPK3-GFP. **d,** Western blot analysis of the necroptosis signaling cascade in HT-29 cells treated with TSZ in time-dependent manner. **e,** Representative confocal images of HeLa cells stably expressing doxycycline (Doxy)-inducible optoRIPK3-HA (red: optoRIPK3-HA, blue: DAPI). Scale bar, 40 μm (left panel). LDH release was measured following TSZ treatment (right panel). n = 3 in each group. Mean ± S.D.; significance was determined by unpaired Student’s *t-*test (right panel). **f.** Representative confocal images of MDA-MB231 cells stably expressing doxycycline (Doxy)-inducible optoRIPK3-HA (red: optoRIPK3-HA, blue: DAPI). Scale bar, 40 μm (upper panel). Western blot analysis showing MLKL phosphorylation and corresponding cytotoxicity, assessed by LDH release, in doxycycline-induced optoRIPK3 cells following TSZ treatment. (middle panel). Western blot analysis showing that light-induced oligomerization of optoRIPK3 results in MLKL phosphorylation and subsequent oligomerization (bottom panel). n = 3 in each group. Mean ± S.D.; significance was determined by unpaired Student’s *t*-test.

**Supplementary Figure S3. |** **Optogenetically activatable RIPK3, lacking RHIM domain, is capable of inducing necroptosis.**

**a,** Schematic representation of RIPK3 mutants with alterations in the N-terminal kinase domain (K50A) and RHIM domain (V467P or 4A) (upper panel). Western blot analysis of 293T cells transfected with Flag-RIPK3 mutants showing that RHIM mutant constructs fail to form oligomer (bottom panel). **b**, Western blot analysis showing the oligomerization of optoRIPK3, which is absent in optoRIPK3(D328), in HeLa cells transfected with optoRIPK3 and optoRIPK3(D328). **c**, Western blot analysis of MLKL oligomerization in HeLa cells stably expressing doxycycline (Doxy)-inducible optoRIPK3-HA or optoRIPK3(D328)-GFP following TSZ treatment. **d,** Representative confocal images of HeLa cells stably expressing doxycycline (Doxy)-inducible optoRIPK3-HA or optoRIPK3(D328)-EGFP (red: optoRIPK3-HA, green: optoRIPK3-EGFP, blue: DAPI). Scale bar, 40 μm. **e,f,** Quantification of cell death in HeLa cells transfected with optoRIPK3 containing either PHR(E281A) or PHR(D387A) under light stimulation. n ≥ 50 cells per group. Mean ± S.E.M.; significance was determined by Mann-Whitney *U* test. **g,** Representative confocal images of HeLa cells transfected with RIPK3(D328)-PHR(E281A)-EGFP or RIPK3(D328)-PHR(D387A)-EGFP, exposed to light at the indicated time points. Scale bar, 20 μm. **h,** Western blot analysis showing MLKL oligomerization in HeLa cells transfected with optoRIPK3(D328) in a light exposure time-dependent manner. **i,** Western blot analysis revealing that optoRIPK3(D328) undergoes RHIM-independent PHR homo-oligomerization in HeLa cells transfected with OptoRIPK3(D328) containing either PHR(E281A) or PHR(D387A). **j,** Western blot analysis showing that optoRIPK3(D328) induces phosphorylation of MLKL under light stimulation, compared to TSZ treatment in HeLa cells transfected with optoRIPK3(D328)-GFP. **k,** LDH release was measured in the cells shown in **j**. n = 3 in each group. Mean ± S.D.; significance was determined by unpaired Student’s *t*-test.

**Supplementary Figure S4. | Optogenetic modulation of MLKL induces its oligomerization.**

**a,** Western blot analysis showing traditional models of RIPK3-dependent MLKL oligomerization in 293 T cells transfected with GFP-MLKL and Flag-RIPK3. **b,** Western blot analysis showing optoMLKL oligomerization independently of apoptosis in HeLa cells transfected with optoMLKL-GFP. **c,** Western blot analysis revealing that optoMLKL oligomerization on 488 nm light, compared to room light, in HeLa cells transfected with optoMLKL-GFP containing either PHR(E281A) or PHR(D387A).

**Supplementary Figure S5. | Optogenetic activation of MLKL induces necroptosis regardless of the presence of RIPK3.**

**a,** Representative confocal images of BT-549 cells stably expressing doxycycline (Doxy)-inducible optoRIPK3-HA or optoMLKL-HA (red: optoRIPK3-HA, green: optoMLKL-HA, blue: DAPI). Scale bar, 20 μm. **b,** Cell viability was measured by crystal violet staining in BT-549 cells treated with or without doxycycline (Doxy) to induce optoMLKL expression, followed by light exposure. n = 3 in each group. Mean ± S.D.; significance was determined by unpaired Student’s *t*-test**. c,** LDH release was measured to assess cell death in BT-549 cells stably expressing doxycycline (Doxy)-inducible optoMLKL, following TSZ treatment or light stimulation. n = 3 in each group. Mean ± S.D.; significance was determined by unpaired Student’s *t*-test. **d,** Western blot analysis of necroptosis signaling cascade in BT-549 cells stably expressing doxycycline (Doxy)-inducible optoRIPK3 or optoMLKL, treated with TSZ or light stimulation. **e,** Representative confocal images of HeLa cells stably expressing doxycycline (Doxy)-inducible optoMLKL-HA (red: optoMLKL, blue: DAPI). Scale bar, 40 μm. LDH release assay was used to measure cell death following light stimulation or TSZ treatment. n = 3 in each group. Mean ± S.D.; significance was determined by unpaired Student’s *t-*test.

**Supplementary Figure S6. |** **Phosphorylation of MLKL is not required for necroptosis induction via optogenetic modulation.**

**a,** Real-time visualization of optoMLKL activation in HeLa cells, revealing membrane blebbing followed by plasma membrane disruption as a result of inducible oligomerization. **b,** BN-PAGE analysis showing MLKL oligomerization and membrane translocation in HT-29 cells treated with TSZ, with or without NSA treatment. **c,** Western blot analysis revealing RIPK3-independent phosphorylation of optoMLKL in BT-549 cells stably expressing doxycycline (Doxy)-inducible optoMLKL in light time-dependent manner. **d,** Immunofluorescence analysis of MLKL phosphorylation in HeLa cells transfected with optoMLKL-GFP upon exposure light stimulation (green: OptoRIPK3-GFP; red: p-MLKL; blue: DAPI). Scale bar, 20 μm. **e,** Western blot analysis showing RIPK3-independent phosphorylation of optoMLKL in the presence or absence NSA and GSK’872. **f,** Western blot analysis showing that the phosphorylation of optoMLKL under light stimulation, which is reversed by treatment with lambda phosphatase (λpp). **g,** Western blot analysis showing phosphorylation of optoMLKL (human) and optoMLKL (mouse) upon light stimulation with or without NSA in 4T1 cells stably expressing doxycycline (Doxy)-inducible optoMLKL. **h,** Western blot analysis showing RIPK3-independent phosphorylation of optoMLKL (mouse) in 4T1 cells stably expressing doxycycline (Doxy)-inducible optoMLKL (mouse) with or without NSA and GSK’872. **i,** Quantification of cell death in HeLa cells transfected with optoMLKL (WT), optoMLKL (T357E/S358D, ED), or optoMLKL (T357A/S358A, AA) following light exposure in a time-dependent manner. n ≥ 50 cells per group. Mean ± S.E.M.; significance was determined by Mann-Whitney *U* test. **j,** Cell viability measured by crystal violet staining in response to 4 hours of light stimulation in HeLa cells transfected with MLKL(WT)-PHR(E281A), MLKL(T357A/T358A, AA)-PHR(E281A), or MLKL(WT)-PHR(D387A). n = 3 in each group. Mean ± S.D.; significance was determined by unpaired Student’s *t*-test. **k,** Oligomerization of MLKL in BT-549 cells transfected with WT, ED, or AA form of optoMLKL following light exposure. Cell lysates were analyzed under non-reducing conditions. **l,** BT-549 cells stably expressing doxycycline (Doxy)-inducible MLKL (WT) or MLKL (1-140). **m,** Cell cytotoxicity in the cells shown in **l** was measured by the Annexin V (AV) and PI-positive cells in doxycycline-induced MLKL expression, assessed using Lionheart FX automated microscopy. n = 6 in each group. Mean ± S.E.M.; significance was determined by ANOVA. **n,** Western blot analysis showing the activation of optoMLKL(WT) and optoMLKL(1-140) upon light stimulation in HeLa cells transfected with optoMLKL (WT) or optoMLKL (1-140). **o,** Cell viability measured by crystal violet staining in the cells shown in **n**. n = 3 in each group. Mean ± S.D.; significance was determined by unpaired Student’s *t*-test.

**Supplementary Figure S7. | DAMPs released upon optogenetic activation of RIPK3 and MLKL.**

**a,** Western blot analysis showing the induction of phosphorylated TRIM28 (S473) during necroptosis. **b,** Inducible DAMPs in HT-29 cells were analyzed by qPCR. n = 3 in each group. Mean ± S.E.M.; significance was determined by unpaired Student’s *t*-test. **c,** Western blot analysis showing that exposure to light induces the phosphorylation of TRIM28 (S473), p38, and p65 in HeLa cells transfected with optoRIPK3, optoRIPK3(D328), or optoMLKL. **d,** Western blot analysis showing optoRIPK3-dependent phosphorylation of MLKL and TRIM28 upon light stimulation in HeLa and MDA-MB231 cells stably expressing doxycycline (Doxy)-inducible optoRIPK3. **e-g**, Inducible DAMPs in the cells shown in **d** were analyzed by qPCR. n = 3 in each group. Mean ± S.D.; significance was determined by unpaired Student’s *t*-test. **h,i,** BT-549 and HeLa cells stably expressing doxycycline (Doxy)-inducible optoMLKL were exposed to light. Inducible DAPMs were analyzed by pPCR. n = 3 in each group. Mean ± S.D.; significance was determined by unpaired Student’s *t*-test. **j,** Silver staining was performed to visualize the total protein released from HeLa, SK-BR3 and BT-549 cells stably expressing doxycycline (Doxy)-inducible optoMLKL. **k,** Released HMGB1 and intracellular levels of HMGB1 were analyzed by western blotting at the indicated time points.

**Supplementary Figure S8. | OptoMLKL activation in patient-derived pancreatic cancer organoid.**

**a,** Experimental timeline for optoMLKL activation in patient-derived pancreatic organoid. **b,** Representative confocal images of immunocytochemical (ICC) staining for phosphorylated MLKL in optoMLKL, or GFP-transduced #70 organoids. Scale bar, 20 μm. **c,** Quantification of the proportion of pMLKL-positive cells in #70 organoids. n = 10 in each group. Mean ± S.E.M.; significance was determined by Welch’s *t*-test. **d,** Quantification of PI-positive cells in #70 organoids. n = 10 in each group. Mean ± S.E.M.; significance was determined by Welch’s *t*-test.

**
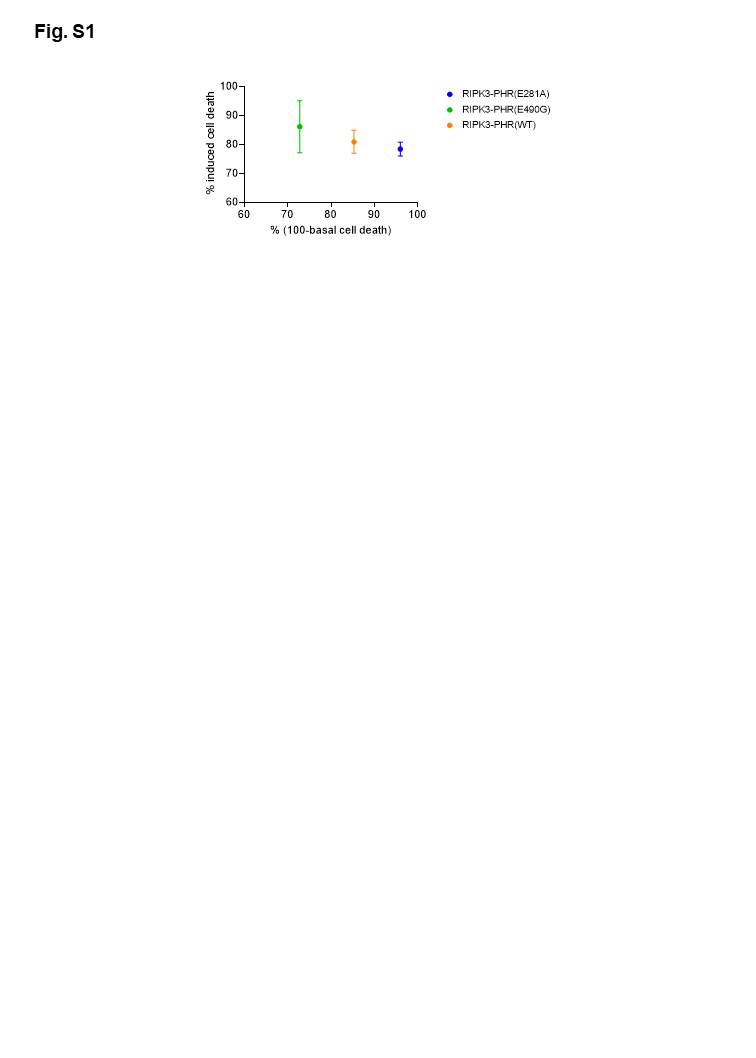

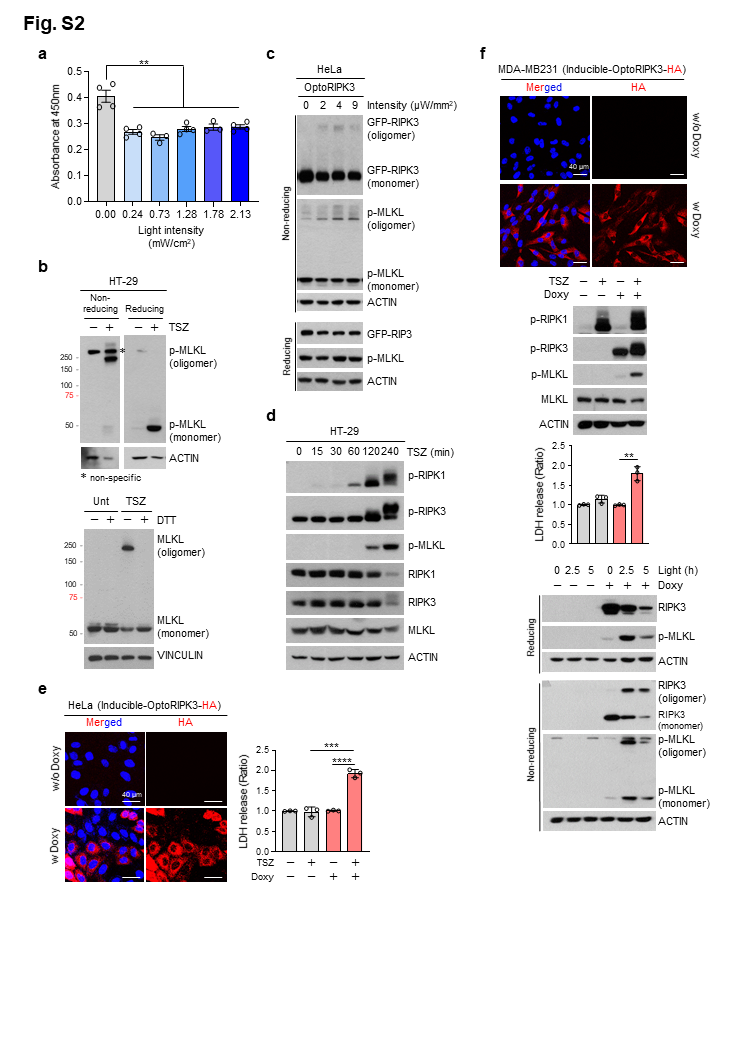

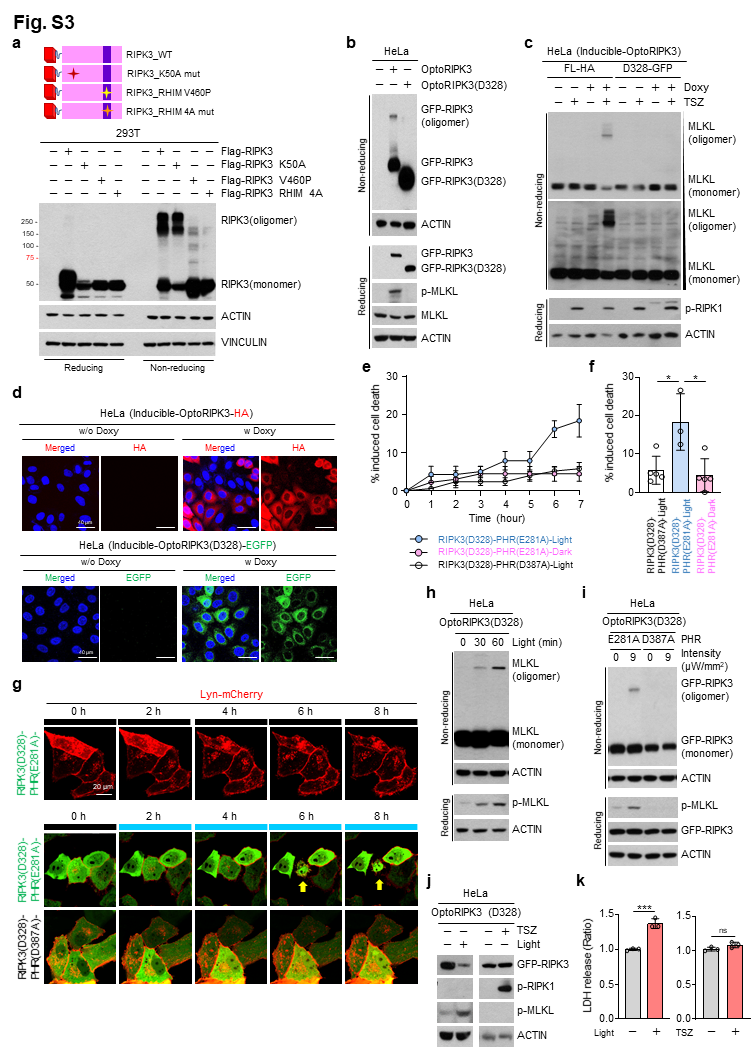

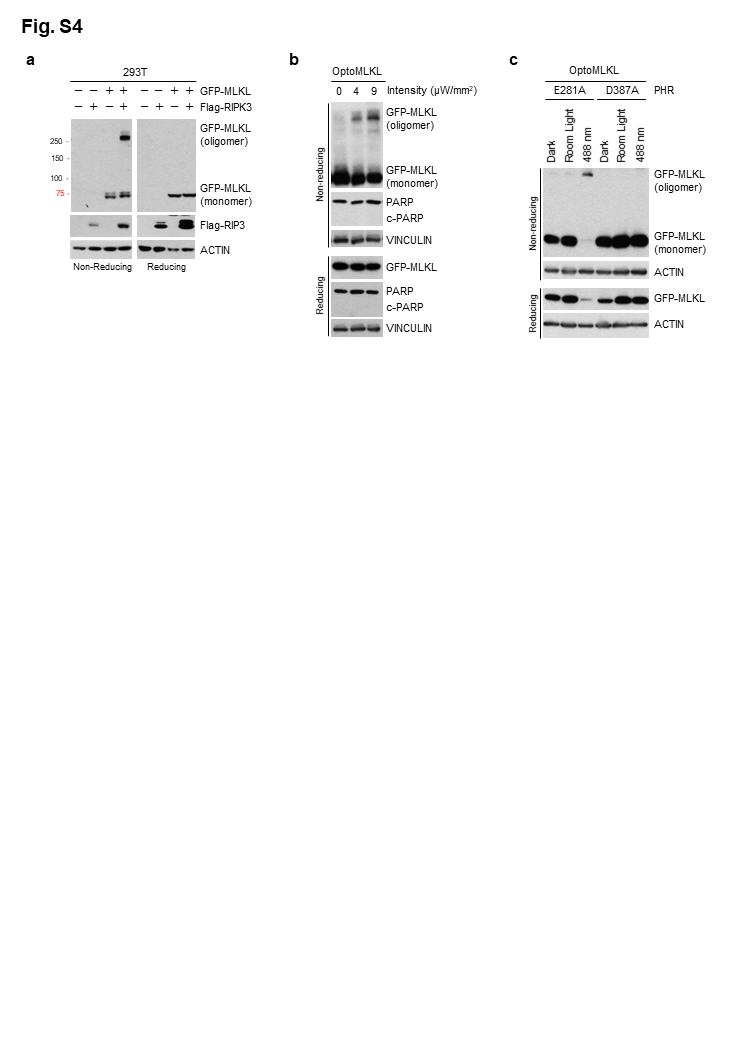

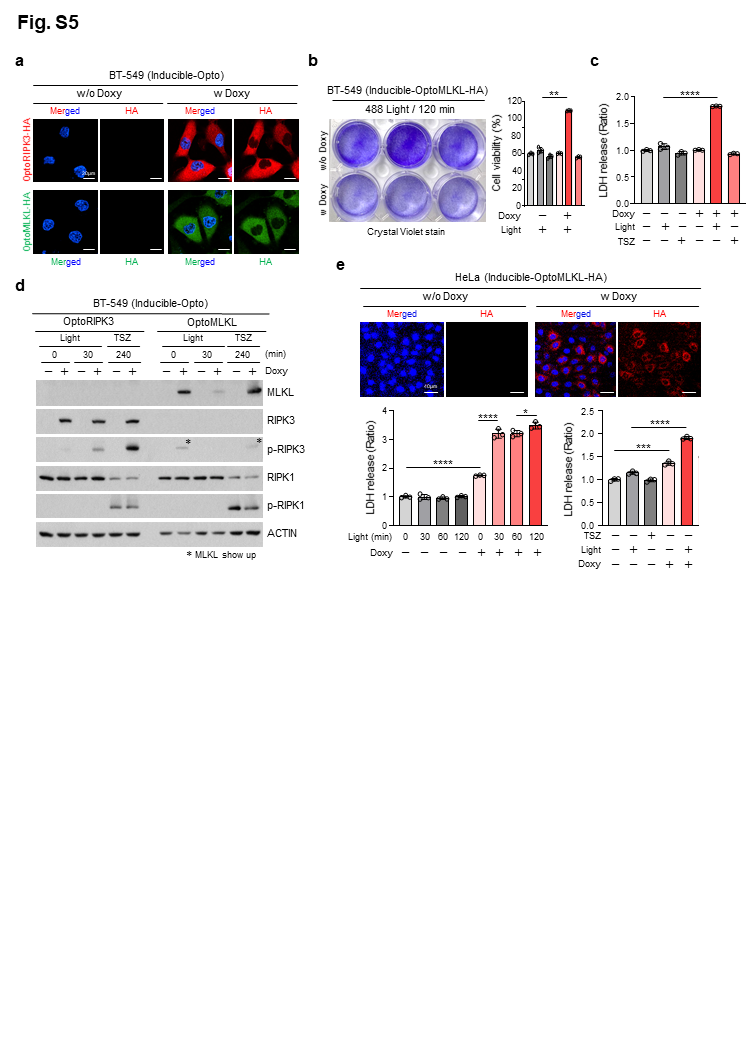

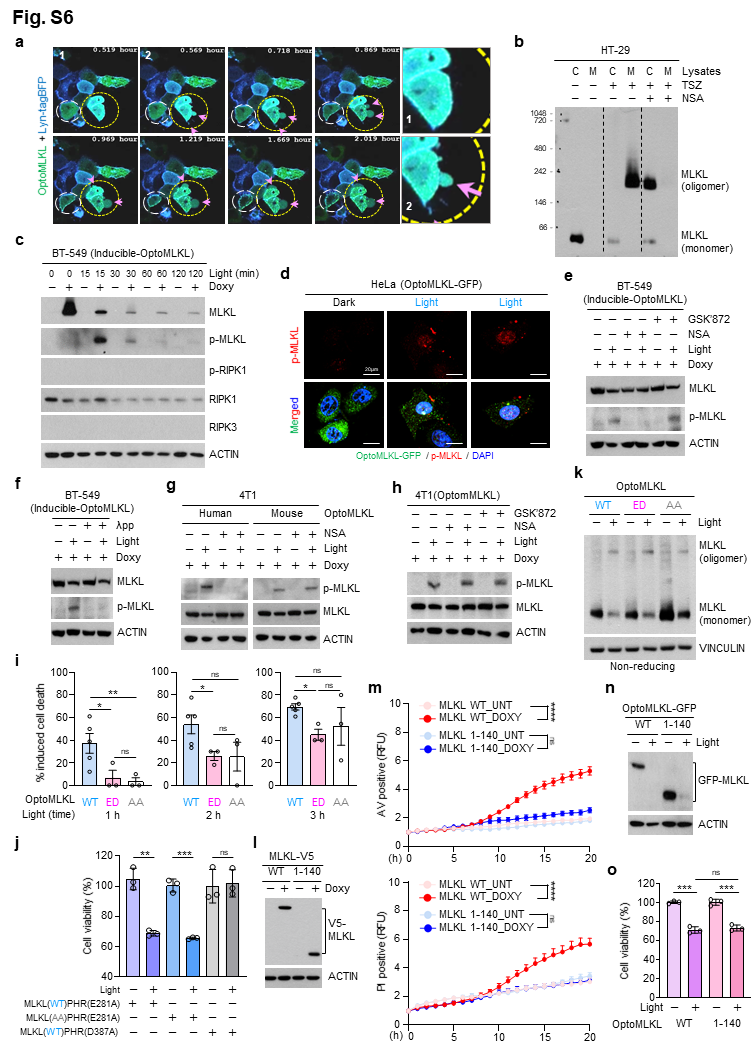

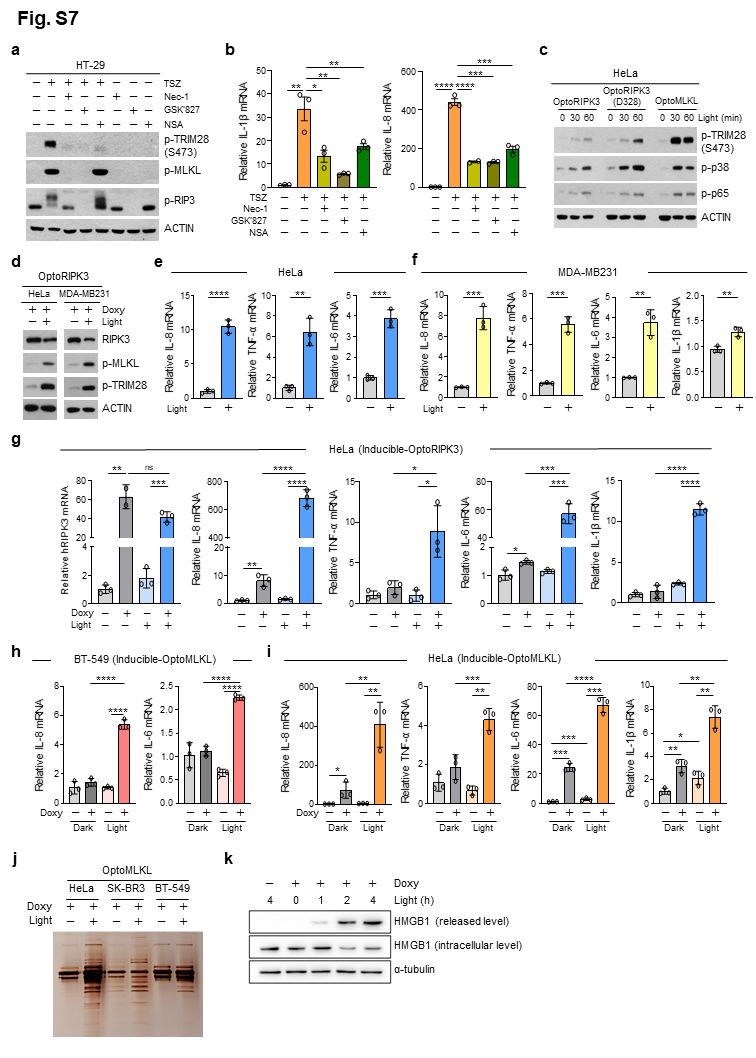

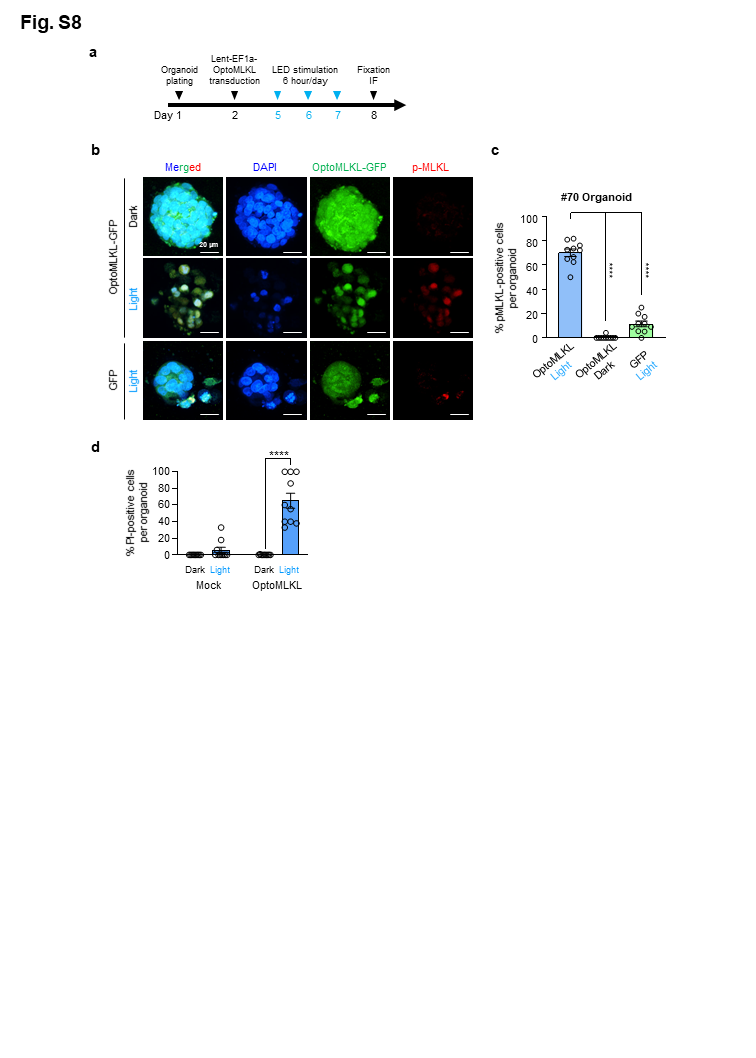
**

**Supplementary data information**

**Table S1.** Clinicopathologic characteristics of patient-derived organoids.

| Sample | Sex | Age | Histological diagnosis | Tumor size | Tumor location | Clinical stage | Clinical TNM stage | Mutation status |
| --- | --- | --- | --- | --- | --- | --- | --- | --- |
| 52 | F | 73 | ADC, P/D | 4.0 cm | Body | IV | T4N1M1 | KRAS G12D |
| 70 | M | 70 | ADC, M/D | 5.0 cm | Tail | IV | T3N2M1 | KRAS G12D |
| 79 | M | 64 | ADC, P/D | 4.4 cm | Body | IV | T4N2M1 | KRAS G12D |

ADC, adenocarcinoma; M/D, moderately differentiated, P/D, poorly differentiated.

**Table S2.** List of primers for plasmid vector construction.

| **Constructed vector** | **Insert** | **Primer sequence** | |
| --- | --- | --- | --- |
| RIPK3-PHR(E281A)-EGFP | RIPK3 | F | GTAGCTAGCCACCATGTCGTGCGTCAAGTTATGGCC |
|  |  | R | GTAAAGCTTCCTTTCCCGCTATGATTATACCAACCCTGT |
| RIPK3-PHR(E490G)-EGFP  RIPK3-PHR(WT)-EGFP  RIPK3-PHR(D387A)-EGFP | PHR(E490G)  PHR(WT)  PHR(D387A) | F | GTAAAGCTTGATGAAGATGGACAAAAAGACCATCGTCTGGT |
|  |  | R | GTAACCGGTATGGCAGCACCGATCATAATCTGCG |
| pLenti-TRE3GV-RIPK3-PHR(E281A)-HA | RIPK3-PHR(E281A) | F | GTAATCGATGCCACCATGTCGTGCGTCAAGTTATGGCC |
|  |  | R | GTAACCGGTGCGTACACGGCAGCACCGATC |
| RIPK3(D328)-PHR(E281A)-EGFP | RIPK3(D328) | F | GTAGCTAGCCACCATGTCGTGCGTCAAGTTATGGCC |
|  |  | R | GTAAAGCTTCCATCCATTTCTGTCCCTCCTTGGCC |
| MLKL-PHR(E281A)-EGFP | MLKL | F | GTAGCTAGCCACCATGGAAAATTTGAAGCATATTATCACCCTTGGC |
|  |  | R | GTAGAATTCTCTTAGAAAAGGTGGAGAGTTTCTTTAAGATTTCATCC |
| MLKL-PHR(E490G)-EGFP  MLKL-PHR(WT)-EGFP  MLKL-PHR(D387A)-EGFP | PHR(E490G)  PHR(WT)  PHR(D387A) | F | GTAGAATTCATATGAAGATGGACAAAAAGACCATCGTCTGGT |
|  |  | R | GTAACCGGTATGGCAGCACCGATCATAATCTGCG |
| pLenti-TRE3GV-MLKL-PHR(E281A)-HA | MLKL-PHR (E281A)-HA | F | GTAATCGATGCCACCATGGAAAATTTGAAGCATATTATCACCCTTGGC |
|  |  | R | GTAGCTAGCGGCGCGCCTTAAGCATAATCTGGA |
| Mouse MLKL-PHR(E281A)-EGFP | Mouse MLKL | F | GTAGCTAGCGCCACCATGGATAAATTGGGACAGATCA |
|  |  | R | GTACCCGGGACACCTTCTTGTCCGTGGATTCTTCAACC |
| pLenti-EF1a-MLKL-PHR(E281A)-EGFP | MLKL-PHR (E281A)-EGFP | F | CCGGTAGCCACCATGGAAAATTTGAAG |
|  |  | R | TATCGCGTCGACTGCAGTTACTTGTACAGCTCGTCCATGCCGAGAG |

**Table S3.** List of primers for real-time PCR.

| **Gene** | **Species** | **Primer sequence** | |
| --- | --- | --- | --- |
| *ACTIN* | Human | F | GGACTTCGAGCAAGAGATGG |
|  | Human | R | AGCACTGTGTTGGCGTACAG |
| *Il-6* | Human | F | TACCCCCAGGAGAAGATTCC |
|  | Human | R | TTTTCTGCCAGTGCCTCTTT |
| *IL-8* | Human | F | TCTGCAGCTCTGTGTGAAGG |
|  | Human | R | AATTTCTGTGTTGGCGCAGT |
| *IL-1β* | Human | F | AAGTACCTGAGCTCGCCAGTGA |
|  | Human | R | TGCTGTAGTGGTGGTCGGAGAT |
| *TNF-α* | Human | F | CAGAGGGCCTGTACCTCATC |
|  | Human | R | GGAAGACCCCTCCCAGATAG |
| *Actin* | Mouse | F | TTGAACATGGCATTGTTACCAACT |
|  | Mouse | R | TCAAACATGATCTGGGTCATCTTT |
| *Tnf-α* | Mouse | F | TATGTCTCAGCCTCTTCTCATTC |
|  | Mouse | R | GTCACTCGAATTTTGAGAAGATGAT |
| *Cxcl1* | Mouse | F | TCCAGAGCTTGAAGGTGTTGCC |
|  | Mouse | R | AACCAAGGGAGCTTCAGGGTCA |
